# Clinical Relevance of Physical Function Outcomes in Cancer Cachexia

**DOI:** 10.3390/cancers16071395

**Published:** 2024-04-01

**Authors:** Lucas Caeiro, Sofia Jaramillo Quiroz, Jenna S. Hegarty, Ellen Grewe, Jose M. Garcia, Lindsey J. Anderson

**Affiliations:** 1 Geriatric Research, Education and Clinical Center, Veterans Affairs Puget Sound Health Care System, Seattle, WA 98108, USA; lucas97@uw.edu (L.C.); sofiaq3@uw.edu (S.J.Q.); jenna.hegarty@icloud.com (J.S.H.); ellieg01@uw.edu (E.G.); jose.garcia@va.gov (J.M.G.); 2 Division of Gerontology and Geriatric Medicine, University of Washington School of Medicine, Seattle, WA 98195, USA

**Keywords:** cancer cachexia, physical function, minimal important difference, minimal important change, minimal clinically important difference, hand grip strength, six-minute walk test, stair climb power, gait speed, quality of life

## Abstract

**Simple Summary:**

Symptom management in the cancer setting focuses on the improvement of the overall quality of life and physical function, particularly for patients with muscle and weight loss, a syndrome known as cachexia, who suffer from low functional ability. Yet, there are no guidelines for measuring or defining low functional ability or standards for identifying meaningful changes in functional ability in response to disease and/or treatment. This unmet need is a major obstacle to developing new/improved therapies for cancer cachexia. This review presents the available evidence for identifying low functional ability and meaningful changes in functional ability in patients with cancer cachexia. Patients with cachexia may display a meaningful reduction in hand grip strength, which may be improved by interventions aiming to increase muscle mass. Future studies should confirm these observations in addition to identifying low functional ability and meaningful changes for other functional outcomes.

**Abstract:**

Managing clinical manifestations of cancer/treatment burden on functional status and quality of life remains paramount across the cancer trajectory, particularly for patients with cachexia who display reduced functional capacity. However, clinically relevant criteria for classifying functional impairment at a single point in time or for classifying meaningful functional changes subsequent to disease and/or treatment progression are lacking. This unmet clinical need remains a major obstacle to the development of therapies for cancer cachexia. This review aims to describe current literature-based evidence for clinically meaningful criteria for (1) functional impairment at a single timepoint between cancer patients with or without cachexia and (2) changes in physical function over time across interventional studies conducted in patients with cancer cachexia. The most common functional assessment in cross-sectional and interventional studies was hand grip strength (HGS). We observed suggestive evidence that an HGS deficit between 3 and 6 kg in cancer cachexia may display clinical relevance. In interventional studies, we observed that long-duration multimodal therapies with a focus on skeletal muscle may benefit HGS in patients with considerable weight loss. Future studies should derive cohort-specific clinically relevant criteria to confirm these observations in addition to other functional outcomes and investigate appropriate patient-reported anchors.

## 1. Introduction

### 1.1. Cancer Cachexia

Supportive and palliative care in the cancer setting has shifted from strictly managing end-of-life needs toward the improvement of symptom burden and quality of life (QOL) across the cancer trajectory, particularly for those with advanced cancer [1,2]. Early supportive/palliative care improves economic outcomes and is strongly recommended by the American Society for Clinical Oncology (ASCO) [3]. One of the primary clinical triggers for supportive/palliative care is functional decline or frailty precluding anticancer therapy [2]. Further, those with advanced cancer are more likely to develop cachexia [4], a “multifactorial syndrome characterized by an ongoing loss of skeletal muscle mass (with or without loss of fat mass) that cannot be fully reversed by conventional nutritional support and leads to progressive functional impairment” [5].

Broadly speaking, cachexia is diagnosed by edema-free weight loss ≥5% in ≤12 months in the presence of underlying illness (body mass index < 20.0 kg/m^2^ is sufficient when weight loss cannot be documented) and absence of starvation, malabsorption, primary depression, hyperthyroidism, and age-related loss of muscle mass [6]. In addition, three or more of the following criteria must be met: (1) hand grip strength (HGS) in the lowest tertile, (2) fatigue, (3) anorexia (limited food intake or poor appetite), (4) low fat-free mass index [lean tissue depletion (mid-upper arm muscle circumference < 10th percentile for age and gender or appendicular skeletal muscle index (kg/m^2^) by dual-energy x-ray absorptiometry: females < 5.45, males < 7.25), or (5) abnormal biochemistry (inflammation [CRP > 5.0 mg/L, IL-6 > 4.0 pg/mL], anemia [Hb < 12 g/dL], or low albumin [<3.2 g/dL]). This consensus statement does not explicitly state the lowest tertile of HGS but refers to two reports; one derived 37 kg as the lowest HGS tertile from Japanese men aged 45–68 living in Hawaii, United States [7]. The other study derived 62 and 41 kg for the lowest tertile of cumulative (both hands) HGS for men and women, respectively, over 65 years of age living in Great Britain [8].

There is also a consensus definition for cancer cachexia diagnosis, including >5% weight loss over six months, 2% weight loss with body mass index < 20, or 2% weight loss with low sex-specific absolute muscularity; it is recommended to obtain a direct measure of muscularity in the presence of edema, large tumor mass, or obesity/overweight [5]. The generally accepted rule for low muscularity is below the 5th percentile, assessed as follows: mid-upper arm muscle area (men < 32 cm^2^, women < 18 cm^2^); appendicular skeletal muscle index (kg/m^2^) by dual-energy x-ray absorptiometry: females < 5.45, males < 7.25; computed tomography 3rd lumbar skeletal muscle index (men < 55 cm^2^/m^2^, women < 39 cm^2^/m^2^); and whole-body fat-free mass index without bone by bioelectrical impedance (men < 14.6 kg/m^2^, women < 11.4 kg/m^2^) [5].

Cancer cachexia is experienced by roughly half of all patients with cancer, is prevalent in up to 80% of advanced tumors, and accounts for 30% of cancer-related deaths [9]. Yet, there is currently no approved treatment in Europe or the United States for cancer cachexia. This is primarily due to a lack of clinically meaningful improvement of physical function or other clinically relevant outcomes in current Phase III trials [10,11,12,13,14,15], despite an improvement of lean mass or body weight compared to the control group in most of these trials. This lack of concordance between muscle mass and function suggests that clinical trials in cancer cachexia should target the improvement of physical function over increasing muscle mass. However, there is no consensus definition available for “progressive functional impairment” in cancer cachexia and, consequently, there are no guidelines for the assessment of physical function at a single point in time or for determining meaningful changes in physical function over time.

Monitoring and addressing deficits in physical function during the clinical management of cancer cachexia may improve QOL, treatment outcomes, and overall survival. In the current clinical practice, physician-rated performance measures (i.e., Karnofsky Performance Status, i.e., KPS, or Eastern Cooperative Oncology Group, i.e., ECOG rating) are utilized to assess physical function; however, these alone do not capture the full spectrum of functional capacity and/or impairment. Therefore, regulatory agencies in the United States and Europe now require clinical trial endpoints that are related to how the patient feels and functions, including physical function, QOL, and survival [16].

### 1.2. Establishing Clinically Meaningful Outcomes

Clinical relevance or importance of outcome performance can be determined via distribution- or anchor-based methods. An anchor-based approach is preferred because the baseline for patient improvement is set by the patient’s own experiential reference measurements. The external criterion in an anchor-based approach can be clinician-rated (i.e., KPS or ECOG rating) or patient-rated (i.e., Functional Assessment of Chronic Illness Therapy, i.e., FACIT). The minimal clinically important difference (MCID) is considered the minimal degree of change or difference in an outcome corresponding to a patient-perceived difference in clinical care or QOL (the anchor) [17,18,19,20] and can be determined cross-sectionally or longitudinally. One common approach is to determine the mean change/difference in an outcome of interest, which reflects one category of change/difference in the anchor (i.e., “able to bathe independently” vs. “requires assistance to bathe”) [21,22]. The MCID can also be estimated by measuring the change/difference in the mean score of the outcome of interest between categories of the anchor (i.e., improved score vs. stable or worsened score) [23]. 

An MCID may also be estimated using an anchor with an established MCID, although previously reported MCID thresholds should be carefully interpreted within the context in which they were ascertained to determine applicability to the cohort in question. It is generally suggested that an MCID should be derived for an outcome of interest from original data if no relevant literature-based MCID is available for the anchor. In this scenario, a dichotomous anchor variable is created from the original data, where patients are categorized “equal to or above” or “below” a predetermined magnitude of change. This dichotomous anchor is then compared to the continuous variable of interest using a receiver operating characteristic analysis [24]. While there is no clear consensus on the analytic approach for the anchor-based strategy, it is advised that the correlation (r) between the change/difference in the outcome of interest and change/difference in the anchor is >0.30, with >0.50 preferred for greater confidence [25], and that the area under the curve from receiver operating characteristic analysis should be ≥0.7 [26].

Distribution-based strategies utilize statistical criteria from outcome scores like effect size, standard error of measurement, and/or fractions of the standard deviation to estimate the minimal important difference (MID), minimal detectable change (MDC), or minimal important change (MIC) [17,18,19,20,27,28,29]. These terms, including MCID, are often referred to interchangeably despite the technical nuances of their derivation. Distribution-based strategies are often considered representative of the degree of change, independent of measurement error. This is a relatively simplistic approach since an external criterion is not required; however, the lack of clinical or patient-perceived validity is also a critical limitation. In addition, a result is produced, which is assumed to have equal magnitude for both directions of change/difference, but this is not always accurate. For example, the MCID for fatigue, as reported by the European Organization for the Research and Treatment of Cancer Quality of Life Questionnaire (EORTC QLQ-C30), was +19.6 points for worsening and −13.3 points for improvement in cancer patients undergoing re-irradiation for painful bone metastases [23]. While the anchor- and distribution-based methods are considered complementary, distribution-based methods should only be used temporarily until data are available to utilize anchor-based methods [20].

### 1.3. Purpose of Review and Summary of Findings 

McDonald et al. stated in 2013 that “the research community has faced the need to identify and develop clinically meaningful outcome measures for use in pivotal therapeutic trials” [30]. Although this was acknowledged a decade ago, the lack of clinically relevant criteria for characterizing functional impairment at a single timepoint or for monitoring functional change subsequent to disease and/or intervention remains a major obstacle to therapeutic development for cancer cachexia. We reviewed the available literature comparing objective physical function between cancer patients with and without cachexia and literature from interventional trials in cancer cachexia reporting a change in objective physical function over time. We then estimated meaningful criteria for functional impairment assessed at a single timepoint and for change in physical function over time.

The most frequently measured physical function assessment in both cross-sectional and interventional studies was HGS. Our findings suggest a potentially important HGS deficit between 3 and 6 kg in cachectic vs. non-cachectic patients, which was repeatedly observed in parallel with worse KPS scores, although this deficit did not reach statistical significance and the relationship between HGS and KPS requires validation. For interventional studies, we observed that longer-duration (three to four months) multimodal therapies that primarily target skeletal muscle may benefit HGS in patients on the higher end of the weight loss spectrum. To improve therapeutic development for patients with cancer cachexia, it is essential to characterize its functional impact. Future studies should derive cohort-specific MCIDs to confirm the current observations, in addition to other functional outcomes, and investigate appropriate patient-reported anchors.

## 2. Materials and Methods

Google Scholar, PubMed, PubMed Central, and clinicaltrials.gov databases were used to search for studies published in English up to 1 August 2023 (the last accession date for clinicaltrials.gov was 8 August 2023). We used the terminology “cancer cachexia” + one of the following: physical function, functional performance, MCID, MID, or MIC. To be selected, the studies had to include (1) human subjects with cancer, (2) cachexia/weight loss as an inclusion criterion (longitudinal studies) or as a grouping/comparison method of cachectic/weight-losing vs. non-cachectic/weight-stable cohorts (cross-sectional studies), (3) measurement and a report of at least one objective physical function assessment, (4) and a report of group means or medians. Studies were excluded (1) for lack of a comparator group, (2) if the study was a sub-analysis or secondary analysis of a larger study included in this review that did not report unique functional outcomes compared to the parent study, (3) if group averages were not reported, and/or (4) if MID or MIC could not be derived for any objective functional outcomes; longitudinal studies were excluded if they were strictly observational.

Three of the most common MID/MIC/MDC estimation equations are listed here [Equations (1), (3), and (4)], although this list is not comprehensive, where SD = standard deviation, CON = control, sqrt = square root, ICC = intraclass correlation coefficient, EXP = experimental, and ANOVA = analysis of variance [30,31]. The equation for calculating ICC using ANOVA terms from the baseline comparison of test–retest reliability for the parameter of interest, presuming lack of bias, is also described [Equation (2)] [32].

[SD of “Time 1” or “CON” parameter] × sqrt [1 − (ICC of “Time 1” test − retest reliability)](1)

[Mean Square Between Subjects − Mean Square Within Subjects]/[Mean Square Between Subjects + Mean Square Within Subjects](2)

[SD of “Time 1” or “CON” parameter]/3 OR(3)

Sqrt [Mean Square Error from ANOVA comparing “Time 1 vs. Time 2” or “CON vs. EXP” parameter](4)

For cross-sectional comparisons, we derived between-group differences (cachectic minus non-cachectic) for outcomes of interest from averages extracted from individual studies and MID estimates using “(non-cachectic SD)/3” [Equation (3)]. For interventional studies, we derived MIC estimates based on two MCID strategies reported in the literature. One strategy utilizes the baseline SD, which is the more commonly reported strategy as described above; here, we calculated MIC-1 “(CON baseline SD)/3.” However, studies utilizing this strategy are primarily single-arm studies, and there is no consensus about whether the SD of baseline or SD of change score is more appropriate in longitudinal studies. Therefore, we also calculated MIC-2 “(SD of CON within-group change score)/3” [Equation (3)], similar to others [25,33].

## 3. Results

Our terminology-based search retrieved 77 publications; 12 review papers were discarded, and 39 original articles were discarded based on *a priori* eligibility criteria. The remaining 26 clinical studies are discussed below in detail.

### 3.1. Functional Impairment from Cross-Sectional Comparisons

We identified seven cross-sectional studies comparing the objective physical function in patients with cancer between those with and without cachexia [34,35,36,37,38,39,40]. Comparisons with non-cancer control groups are provided in some of these studies but are not discussed herein as that comparison was beyond the scope of this review. Study details, including group means extracted for outcomes of interest, our calculated differences between the extracted group averages, and our derived MID estimates, are provided in Table 1. The studies utilized fairly consistent cachexia definitions and primarily consisted of solid tumor cohorts. All seven studies reported HGS; three reported habitual gait speed, and two reported the 6-minute walk test (6MWT), isometric knee extension strength, and the 5-times sit-to-stand (5STS) test. The following outcomes were only reported in one study each: stair climb power (SCP), maximal strength of upper and/or lower body muscles, timed physical performance test, get-up-and-go, lower limb extensor power, maximal gait speed, and accelerometry-based physical activity.

#### 3.1.1. Objective Physical Function

Among the seven studies reporting HGS, the results from Burney et al. and the male-only results from Dolin et al. were omitted due to the presentation of cumulative HGS of both hands and due to a sample size of <8 per group, respectively [35,37]. For the remaining six HGS assessments, the difference in group means ranged from 0.7 kg greater to 6.6 kg lower, HGS in cachexia vs. non-cachexia and the MID estimate ranged from 1.6 to 4.1 kg. Three of the six studies reported significantly lower HGS in cachexia, which were the three studies with the largest magnitude of group difference (5.3 to 6.6 kg lower) [36,38,39]. These studies, in addition to the female group in Stephens et al., which displayed 5 kg lower HGS [40], were the only studies to display group differences larger than the respective MID estimates. However, HGS may have only been lower in the cachexia group reported by Ohmae et al. due to a numerically larger proportion of females in the cachexia (15%) vs. the non-cachexia (30%) group, but this difference in proportions was not significant.

Three studies assessed some form of quadricep/leg strength, which was numerically lower with cachexia, and the between-group differences were larger than their respective MID estimates, indicating that these magnitudes of reduced quadricep/leg strength in cachexia may have potential for clinical importance [34,39,40]. While these differences did not typically reach statistical significance, it was statistically significant for strength as a percentage of body weight reported by Ohmae et al., likely due to a larger (numerically, but not significantly, as noted above) proportion of cachectic than non-cachectic females, and it was also significant for absolute, but not relative, strength for females, as reported by Stephens et al. [39,40].

Habitual gait speed averaged 1.0 to 1.2 m/s for all groups in the three studies reporting this outcome [37,38,39]. Two of these studies reported a significant between-group difference of roughly 0.2 m/s slower with cachexia [38,39], which nearly exceeds the MCID range of 0.1 to 0.2 m/s previously reported from mixed cohorts of community-dwelling older adults and/or older adults with mobility disability or chronic diseases/conditions [41,42]. This between-group difference was also larger than the derived MIDs, indicating this may be an important impairment in normal gait speed in cachexia. 

Two studies reported 5STS, which averaged about 10 seconds in all groups, except for the non-cachectic group from Ohmae et al., which was around 2 seconds faster, a difference that was larger than the derived MID for that study; an MID could not be derived for the other report [37,39]. The 6MWT was also assessed in two studies which reported inconsistent group averages, especially for the non-cachectic groups. One study reported a better performance of 41 m while the other reported a worse performance of 31m in cachectic patients; these group differences were nearly equal to their derived MIDs and were not statistically significant in either study [37,38]. The discrepancy may be due to differences in population characteristics (i.e., one included only colorectal cancer, while the other included advanced cancer with 16% colorectal tumors), sample size, and/or outcome variability.

Receiver operating characteristics from our previous report indicated that upper body strength and SCP may be useful assessments (area under the curve > 0.7), with potential cut-points displaying high sensitivity and specificity for identifying patients with cachexia [34]. The between-group differences in upper body strength and SCP in that study were also two to four times greater than the corresponding MID estimates. These were only statistically significant for chest press strength and SCP, which may be due to reduced statistical power in the original analysis, which utilized ANOVA for comparison to a third group (non-cancer control). Stephens et al. also reported a group difference in leg power, both absolute and relative to body weight (relative function is often used as an indicator of muscle quality), revealing lower power in males with cachexia, which was larger than the estimated MID [40]. This was not observed for females and was not statistically significant for either; however, their original analysis also utilized ANOVA for comparison to a non-cancer control, which may have reduced their statistical power.

#### 3.1.2. Subjective Physical Function

Five studies reported subjective measures of physical function and/or other QOL parameters with strong physiological relevance to physical function (i.e., fatigue, frailty, etc.; we did not report an exhaustive list of all subjective measures assessed by each study). All five reported KPS [34,35,36,38,40], three reported ECOG, and two reported FACIT-Fatigue (-F) and the Anderson Symptom Assessment Scale (ASAS) [34,35]. One study reported the EORTC QLQ-C30, Patient-Generated Subjective Global Assessment (PG-SGA), and EuroQol 5-Dimension 5-Level assessment (EQ-5D-5L) [36,38,40]. With the exception of Hadzibegovic et al., who reported an overall worse performance rating for all patients, average KPS ranged from 77 to 90 for cachexia and from 84 to 100 for non-cachexia, and average ECOG ranged from 0 to 1 for all groups [34,35,36,40]. Average KPS most often ranged from 10 to 14 points worse in cachexia than non-cachexia, while ECOG scores were similar between groups, ranging from 0.1 better to 1.0 worse in cachexia. MID estimates ranged from 3 to 7 for KPS and 0.2 to 0.4 for ECOG. 

However, ECOG and KPS self-contain clinically important standards pertaining to one category change in performance rating: 10 points for KPS and 1 point for ECOG. Our observations may suggest that KPS is a more sensitive index than ECOG for differentiating between patients with or without cachexia. The detriment in HGS ranged from 3 to 6 kg for cachexia in the four studies reporting ≥10-point lower KPS for cachexia, suggesting that a meaningful impairment in HGS may fall between 3 and 6 kg below the cohort-specific non-cachectic average. One of these four studies is a previous report from our laboratory, where HGS was not significantly different between groups, although as noted in Table 1, we observed a 1-point lower (worse) ECOG rating with cachexia [34]. In addition, FACIT functional well-being and ASAS Total were worse in cachexia according to our MID estimates, although not statistically significant. Correlations between KPS and HGS were not performed by the studies reviewed here; therefore, the potential relationship between HGS and KPS will need to be directly confirmed by future studies or by additional analyses, like correlations, on currently available data.

Receiver operating characteristics also indicated that a PG-SGA score of >6.5 may be useful for identifying cachexia [36]. Hadzibegovic et al. conducted the only study to correlate objective physical function and patient report outcomes and observed a direct association between HGS and EQ-5D-5L score (*r* = 0.32, *p* < 0.001 for cachectic and non-cachectic patients combined), which may indicate this survey has potential to serve as an anchor for determining the MCID of HGS in cachectic individuals with advanced solid tumors [38]. Considering the EQ-5D-5L is not widely used in this field, we encourage investigators to explore more commonly utilized patient-reported outcomes to improve the characterization of functional impairment and clinically meaningful changes in cancer cachexia.

### 3.2. Functional Changes from Interventional Studies

We identified 19 interventional trials reporting a change in objective physical function that either explicitly recruited patients with cancer cachexia or utilized entry criteria to target individuals with a high likelihood of having/developing cancer cachexia (Table 2). Within-group change for outcomes of interest, between-group differences in outcome change, and our derived MIC estimates based on the corresponding control/reference group are provided in Table 2. It is important to note that an intervention benefit may be observed either by the mitigation of outcome worsening or by enhancement of outcome improvement relative to the within-group change of the reference group. 

Cohorts were mostly comprised of advanced-stage cancer from any tumor site, any solid tumor, or a specific lung tumor; all but two studies exclusively enrolled patients with specific cachexia entry criteria. The most frequently reported objective functional outcome was HGS, which was reported in 17 studies: one reported HGS separately for the dominant and non-dominant hand, one only reported non-dominant HGS, and another reported the mean for both hands. However, the majority did not specify which hand was used. The 6MWT, SCP, and daily step count were reported in two studies, and leg extension torque/power, Short Physical Performance Battery (SPPB), and habitual gait speed were reported in one study.

Seventeen studies reported subjective measures of physical function and/or other QOL parameters with strong physiological relevance to physical function (i.e., fatigue, frailty, etc.; we did not report an exhaustive list of all subjective measures assessed by each study). Parameters assessing physical function either exclusively or partially included ECOG reported in five studies, FACT-G functional well-being in two studies, and SF-36 physical function, KPS, and PG-SGA reported in one study each. Fatigue was assessed with the FACIT-Fatigue sub-scale, FACIT-F Total, or Multidimensional Fatigue Inventory in two studies each, and ASAS/ESAS fatigue or Fatigue Severity Scale in one study each. Overall QOL and/or cachexia-specific QOL was assessed by the FAACT-Anorexia/Cachexia component or -Total score in five studies, EORTC QLQ-C30 Global QOL or -Total score in four studies, and FACT-G Total or ASAS Total in one study each.

#### 3.2.1. Interventions Targeting Skeletal Muscles

Six interventions aimed to increase muscle mass by administering androgen receptor agonists [43,45], anabolic nutrition/supplementation [47,48], or multi-modal treatments involving nutrition and anti-inflammatories with or without exercise [44,46]. The primary component(s) of these interventions upregulate skeletal muscle protein synthesis and downregulate protein degradation by stimulating the androgen receptor (enobosarm and testosterone), increasing intramuscular leucine or carnitine concentrations (high protein supplements, particularly whey protein), improving ATP-cycling (creatine), and mechanical signaling (exercise, particularly resistance training). Objective physical function was assessed by HGS in five studies, while 6MWT, gait speed, SCP, leg extension torque and power, SPPB, and/or physical activity (daily step count) were measured in one study. In the studies measuring HGS, MIC-1 ranged from 2.7 to 4.2 kg. The within-group change ranged from −0.4 to +3.0 kg for EXP and −1.1 to +0.1 kg for CON, although it did not reach significance for either group in any report. MIC-2 ranged from 0.3 to 2.7 kg, and the between-group difference ranged from 0.3 to 4.1 kg higher HGS in EXP than CON, which were mostly not statistically significant.

Dobs et al. compared 16 weeks of 1 or 3 mg/d of enobosarm, a selective androgen receptor modulator, to placebo for improving total lean mass [43]. SCP significantly improved in both treatment arms with no change in placebo; no between-group differences were reported. The 1-mg group also significantly improved QOL and fatigue. Lean mass significantly improved in the 3 mg group compared to placebo and displayed a between-group trend in the 1-mg group [43]. Between-group differences in physical function change were not reported. This provides another example of discordance between muscle mass and function, further supporting the premise that clinical trials in cancer cachexia should target the improvement of functional outcomes over increasing muscle mass. 

A small study of intramuscular testosterone or placebo for seven weeks revealed greater (but not statistically significant) improvements in leg extension torque/power, SPPB, and subjective function [45]. MICs could not be derived from the information provided, but SPPB improved by an additional 1.1 point beyond that observed with placebo, which is greater than the 1-point MCID previously reported in a large cohort of community-dwelling older adults and older adults with mobility disability or subacute stroke survivors [41].

In addition, three months of nutritional counseling with whey protein induced significantly greater HGS improvement relative to counseling alone in patients with considerable weight loss (≥10% in six months) [47]. This was the only study to report a significant between-group difference, which was also larger than the derived MIC-2, but this impact on HGS was not observed with a concomitant improvement in QOL. Another study tested the impact of creatine, which is purported to improve ATP-cycling [58] or placebo for two months, and reported no change in HGS for either group [48]. 

The pre-MENAC study compared six weeks of exercise, nutritional support, and anti-inflammatory medication to usual care and reported no between-group difference for change in HGS, 6MWT, daily step count, PG-SGA, or fatigue [44]. Fatigue increases were larger than the derived MIC-1 in EXP and 6MWT, and daily step count increases were larger than the derived MIC-1 in CON, favoring usual care. However, no within-group changes were statistically significant as this was a small feasibility study, which was not powered to detect changes in all secondary outcomes. 

Lastly, four months of carnitine, antioxidants, and the appetite stimulant megestrol acetate administration were compared to megestrol acetate alone in patients with considerable weight loss (≥5% in three months) [46]. The carnitine-containing group (EXP) displayed numerically greater HGS change and significantly improved fatigue and QOL relative to megestrol alone. The within-group change for HGS in EXP was also larger than the derived MIC-1, although MIC-1 was based on the megestrol group baseline SD; yet, megestrol alone is not a true control for comparison [46].

Overall, these interventions did not display a high degree of success in improving physical function. The most consistent impact was observed on HGS after three to four months of whey-containing supplementation or carnitine/antioxidant/megestrol in patients with considerable weight loss [46,47]. Improvements in HGS for these two studies were 2.3 [47] and 4.1 kg [46] higher than their respective comparator group changes, which were greater than the corresponding MIC-2 estimates, but only one reported a significant between-group difference [47]. The other study displayed a within-group HGS improvement (3 kg) greater than the MIC-1 estimate (2.7 kg), concurrently with a statistically significant between-group improvement in QOL and fatigue [46]. 

#### 3.2.2. Interventions Targeting Appetite

Synthetic progestins like megestrol acetate and ghrelin analogs like anamorelin and macimorelin stimulate appetite by promoting neuropeptide Y secretion from the hypothalamus. Mirtazapine is an antidepressant currently recommended by the National Comprehensive Cancer Network for treating depression and anorexia in cancer cachexia, although it only recommends mirtazapine for depression once life expectancy reduces to months or weeks or less [59]. While its exact appetite-stimulating mechanisms are unclear, it is purported to exert an analgesic and antiemetic effect through the antagonism of central 5HT_2_/5HT_3_ receptors and has recently been shown to increase ghrelin levels in a non-cancer setting and improve caloric intake and gastric motility in patients with cancer-related anorexia [60,61,62]. Similarly, ASCO recently revised its categorization of the anti-depressant olanzapine from “no recommendation” to “moderate in favor” based on its efficacy for improving appetite and weight, although efficacy was primarily tested in patients with lung/gastrointestinal cancer and chemotherapy-induced anorexia [63].

Other “moderate in favor” recommendations from ASCO for improving appetite and body weight in cancer cachexia include dietary counseling, progesterone analogs, and corticosteroids. The society indicated “no recommendation” due to low levels of evidence for the efficacy of anamorelin, olanzapine, and NSAID interventions (note: this is not an exhaustive list of the recommendations) [64]. The National Comprehensive Cancer Network makes specific recommendations, which are variable based on life expectancy, for the use of megestrol acetate, olanzapine, or dexamethasone to improve appetite and metoclopramide to improve satiety [59]. Eight studies in the current review administered appetite stimulants, all of which reported HGS. The within-group change ranged from −1.6 to +3.9 kg in EXP and −1.6 to +6.8 kg in CON. The between-group difference in change ranged from 3.2 kg lower to 0.9 kg greater HGS in EXP, and MIC-2 ranged from 0.3 to 1.9 kg. 

Megestrol acetate and mirtazapine were each administered as appetite stimulants to patients with cancer cachexia. Mirtazapine was unsuccessful at improving HGS or appetite compared to placebo [52]. The within-group change did not exceed MIC-1 for either group, but the between-group difference numerically exceeded MIC-2 in favor of placebo, although none of these comparisons reached statistical significance. Megestrol, in combination with carnitine and celecoxib (EXP), numerically improved physical activity by nearly 1,000 additional daily steps than carnitine and celecoxib alone (CON), although the between-group difference was only a trend (*p* = 0.086) [12]. In that study, EXP and CON significantly improved 6MWT (45 and 53 m on average, respectively) and fatigue, but between-group differences were not significant. However, the efficacy of carnitine and celecoxib with or without megestrol acetate cannot be determined without usual care or another appropriate control group for comparison. In addition, MICs could not be derived from the information provided, but 6MWT improvement for each group was greater than the widely reported MCID of 30.5 m in adults with chronic pathology [26]. Fatigue improvement for both groups was also consistent with EXP (carnitine + celecoxib + lipoic acid + carbocysteine + megestrol acetate) in Maccio et al. [46] for the Multidimensional Fatigue Symptom Inventory (on average, 6.4 to 8.8 points across both studies). 

In two separate studies, megestrol was administered over two months with or without celecoxib [50] or thalidomide [51]. HGS numerically improved after megestrol administration, with or without celecoxib, by the largest magnitude of any observation reported here (3.9 and 6.8 kg, respectively) but was only significant for megestrol alone [50]. In that study, both groups improved QOL, but only megestrol alone significantly improved ECOG score. In another study, the addition of thalidomide to megestrol acetate improved HGS, ECOG, and fatigue, reaching within- and between-group significance for all three outcomes [51]. The between-group difference was larger than the derived MIC-2 for all three, although the within-group change was smaller than MIC-1 for all three. However, the true efficacy of thalidomide cannot be determined without usual care and a thalidomide-only or another appropriate control group for comparison.

Three randomized controlled trials examined the efficacy of the ghrelin agonist anamorelin. Garcia et al. administered 50 mg/d for 12 weeks in a Phase II trial [15], ROMANA 1 and 2 administered 100 mg/d for 12 weeks in two parallel Phase III trials [14], and ROMANA 3 extended ROMANA 1/2 for an additional 12 weeks [11]. Within-group change in HGS for these three trials ranged from −1.6 to +1.6 kg in EXP and −1.6 to +0.7 kg in CON. The between-group difference in HGS change ranged from 0.6 kg lower to 0.9 kg higher HGS with anamorelin than placebo, which was not statistically significant in any of these three trials, nor was there a potential clinical importance based on our derived MICs. Overall QOL, but not fatigue, improved with anamorelin in the two 12-week trials but was not different between groups at the end of ROMANA 3. Another ghrelin agonist, macimorelin, was administered for one week in a pilot study; the trial ended early due to low recruitment; so, patients receiving either dose were combined for comparison [49]. HGS and SCP change did not differ from placebo, but the between-group difference for FACIT-Fatigue sub-score (−4 points) indicated an improvement with macimorelin. The magnitude of this within-group change was greater than the previously reported MCID of 3 points in patients undergoing fatigue-inducing chemotherapy [65]. 

Appetite stimulants displayed minimal efficacy as an overall category for improving physical function, primarily measured by HGS here. Ghrelin agonists did not impact HGS, nor were the changes greater than the derived MICs [14,15]. Megestrol may have displayed potential for HGS and QOL benefit, with better efficacy alone than in combination with celecoxib [50] but less efficacy alone than in combination with thalidomide [51]. 

#### 3.2.3. Immunomodulators and Oral Supplements

ASCO’s recommendation for the TNF inhibitor class of immunomodulators is “moderately against”, although “no recommendation” is specifically indicated for thalidomide, an anti-inflammatory primarily known as a TNF inhibitor [64,66]; “no recommendation” is also specifically indicated for vitamins, minerals, and dietary supplements [64].

Although Wen et al. observed that thalidomide with megestrol acetate improved HGS, ECOG, and fatigue, two months of thalidomide alone worsened HGS compared to placebo, although not statistically significantly [57]. QOL was not assessed, and MIC could not be derived from the information provided. Another small study observed that 12 weeks of thalidomide plus cinobufagin significantly improved HGS and QOL relative to cinobufagin alone, and within-group changes in EXP were larger than their respective MIC-1 estimates [56]. Infliximab, a TNF antibody, induced significant amelioration of 6MWT decline with 5 mg/d, but not 3 mg/d, compared to placebo [54]. The between-group difference in total FACIT-F was larger than derived MIC-2, suggesting that the 5-mg group may have also displayed a meaningful benefit on overall QOL compared to placebo. However, this was a small study, and the between-group difference did not reach statistical significance, which will require future validation in a well-powered trial. However, eight weeks of infliximab + docetaxel significantly worsened fatigue and global QOL compared to placebo + docetaxel in patients with NSCLC [67]. This trial was not included in the current review due to a lack of objective functional endpoints, but it was highly influential in ASCO’s recommendations as there were no group differences in survival or tumor response, and the trial closed early due to a lack of efficacy for the primary outcome, which was ≥10% weight gain [64].

Another small study compared two months of herbal supplements to a placebo and reported greater improvement in HGS relative to placebo [55]. An MIC could not be derived based on the available information, and the impact on HGS was not observed in parallel with improved QOL. A different small study assessed the safety and tolerability of targeted medical nutrition compared to an isocaloric supplement control but observed no difference between groups for change in HGS or daily step count [53]. The lack of difference may be due to the small sample size, considering the relatively large between-group differences, but this difference was only larger than the derived MIC-2 for non-dominant HGS and step count. Data supporting the potential for immunomodulators, particularly TNF inhibitors, which appear to be the most commonly tested, or oral nutritional supplements to improve physical function or ameliorate functional decline in cancer cachexia, are widely inconsistent and limited to small studies that require further validation.

## 4. Discussion

Cachexia is one of the most underrecognized triggers of initial functional decline; yet, it is present in roughly half of all patients with cancer and up to 80% of those with advanced tumors [9]. Cachexia also accounts for 30% of cancer-related deaths and includes “progressive functional impairment” as a defining feature [9]. The collaboration between all healthcare providers, including nurses, dietitians, physical therapists, and physicians (primary care, oncologists, endocrinologists, and supportive/palliative care specialists), for functional status management in cancer cachexia is critical. The standardization of physical function assessment and, subsequently, of functional decline and improvement, remains a major unmet clinical need in the cancer cachexia setting. 

Criteria are lacking for characterizing presentation, or risk, for functional impairment at any given point in time, such as that provided for sarcopenia, wherein HGS and 5STS are used for diagnosis (which require confirmation via the direct measurement of muscularity), while the timed up-and-go, 400-m walk, SPPB, or habitual gait speed alone can be used to determine sarcopenia severity [68,69]. As with cancer cachexia, parameters for clinically meaningful improvement or decline in these functional outcomes remain an unmet need [70]. The current bottleneck in criteria development is upstream of healthcare providers given that researchers and regulators have collectively failed to deliver an intervention that is approved for treatment. Multimodal treatment, particularly the combination of medication, exercise/physical activity, and diet/nutrition, has the highest likelihood for the successful amelioration of cachexia syndrome; yet, these complex treatments introduce equally complicated regulatory and clinical considerations for trial design [71]. Little progress has been made over the past decade toward the resolution of these issues, but advocacy efforts and working groups continue to bridge the gaps between key stakeholders, with the central tenet of focusing on endpoints that are meaningful to patients [72,73,74,75]. The integration of MCIDs into these discussions and, ultimately, into clinical trial design, will significantly improve the relevance of functional endpoints, although there are no criteria for establishing the magnitude of functional change that is considered a clinically meaningful response to disease progression and/or therapeutic/supportive intervention. This review focused on clinically meaningful criteria for evaluating functional impairment at a single timepoint and changes in function over time. Future multimodal treatment studies will need to test the applicability of MCIDs derived from unimodal interventions.

### 4.1. Functional Impairment at a Single Point in Time

Reduced quadricep strength and habitual gait speed with cachexia were observed in a small number of studies, but the most frequently reported and consistently supported observation of worse functional performance with cachexia was HGS. This outcome was reported in all seven cross-sectional studies identified here. An MID could be derived in five of them, ranging from 3.3 to 4.1 kg. In four of these studies, the cachectic group displayed a lower (worse) KPS score of ~10 or more points (corresponding to at least one category change in performance rating) than the non-cachectic group; the between-group difference in HGS ranged from 3 to 6 kg lower in the cachectic group in these four studies. This observation supports the potential importance of a cachexia-related HGS deficit in this range. However, its clinical relevance requires verification with anchor-based methods. It would be optimal to also compare non-cancer controls to better understand the interplay between the effect of cachexia from the effect of the cancer itself. As noted in Section 3.2 and Table 1, this comparison was beyond the scope of the current review as only a few studies included non-cancer controls. In addition, criteria for unimpaired HGS cannot be reliably determined from the variable cohorts included here. 

However, when tested for association with weight loss in 1500 patients (40% cancer patients) recently admitted to the hospital, HGS displayed similar results to those observed here [76]. In that study, HGS was reduced in weight-losing patients (2.7 to 3.7 kg in females and 3.6 to 6.7 kg in males) which was particularly similar to our observations in cachectic males. Further, the application of our MID estimation method to the weight-stable group in that study produces a MID-1 estimate of 2.6 kg for females and 3.8 kg for males, which is also similar to our findings, and suggests that our observations may be generalizable to a broader setting. In another review, the MCID for HGS change was reported across different cohorts ranging from patients with myotonic dystrophy (0.04 kg) to those undergoing surgical repair of radial fracture (6.5 kg) [24]. This supports the premise that MCIDs should not be broadly generalized without verification due to disease/treatment-specific impacts on outcomes of interest and/or variability in MCID derivation [77]. 

### 4.2. Important Functional Change over Time

Several molecular mechanisms have been shown to be altered in animal models of cancer cachexia, including a decline in protein synthesis and an increase in protein degradation, among others [66]. Addressing this issue might require skeletal muscle-targeting interventions with the potential to offset these dynamics. A possible solution could involve the employment of multimodal approaches like exercise, in addition to pro-anabolic pharmaceuticals or dietary supplements [78].

The most consistent benefit for physical function was observed on HGS after three to four months of whey-containing supplementation or carnitine/antioxidant/megestrol in patients with considerable weight loss [46,47]; the whey-containing study also improved QOL [46]. HGS improvement relative to the comparator group was 2.3 [47] and 4.1 [46] kg in these studies, indicating that an important magnitude of improvement may lie within this range, although only the carnitine-containing study displayed a significant between-group difference [47]. Additionally, Enobosarm (1 mg/d) improved SCP and QOL, but preliminary results from two Phase III trials in patients with non-small cell lung cancer and cachexia reported improved muscle mass, with no impact on SCP [10]. We expected an improvement across numerous functional outcomes after three to four months of any anabolic intervention [79,80], which were not consistently observed here. 

Megestrol acetate is approved for the treatment of cachexia in acquired immunodeficiency syndrome [81], but it is reportedly ineffective for improving anorexia/cachexia symptoms in advanced cancer [82]. We observed a similar lack of efficacy in the current review, where megestrol did not significantly enhance the impact of carnitine plus celecoxib on HGS, 6MWT, daily step count, or fatigue [12], and mixed results were reported for HGS with megestrol alone compared to the combination with celecoxib or thalidomide [50,51]. Anamorelin did not significantly improve HGS either [11,14,15], despite significant QOL improvement compared to placebo [14,15]. Moreover, two randomized controlled anamorelin trials in Japan reported a lack of significant HGS change vs. placebo [83,84]. One of those trials also reported a lack of 6MWT change, implying that HGS is not an insufficient functional assessment tool but that anamorelin may not be successful at eliciting functional change in cancer cachexia [83]. These anamorelin studies from Japan were not included in the current review because they reported least square means; so, between-group differences could not be extracted.

Immunomodulators and oral supplements reviewed here showed some benefits for physical function. Two studies reported significantly greater HGS improvement of ~1 to 3 kg [55,56] and one reported significant attenuation of 6MWT decline by ~26 m after infliximab [54] compared to the control. Cancer treatment-related MIC for 6MWT was reportedly between 22 and 42 m for patients with lung cancer of any stage undergoing various regimens, 40% of which self-reported weight loss of an unknown amount [85]. The within-group changes after infliximab or a placebo observed in the current review were larger than 42 m, but the treatment-related MIC range from the prior study was reported after only 10 days, while infliximab was administered for eight weeks [54]. Other chronic disease settings reported a similar 6MWT MIC (14 to 38 m) as the current observations [26,86,87,88]. However, all immunomodulator and oral supplementation studies reviewed here require future validation from adequately powered trials for further recommendation.

## 5. Limitations

There are some important limitations to consider for the application of these findings. Here, we used descriptive statistics from published studies to derive estimates of clinically meaningful differences/changes, which may be different if derived using individual patient data. We also compared between-group differences in change from interventional studies to our derived MIC-2 (from SD of CON change); however, a responder analysis would be the preferred approach for comparing between-group differences in change [89]. We can only state whether we observed concomitant differences/changes in function and QOL, we cannot verify correlations or area under the curve values unless reported by individual studies. In addition, cohorts that predominantly developed sarcopenic obesity rather than cachexia, like breast and prostate cancer, were excluded from this review due to our eligibility criteria; however, an agreement of functional impairment thresholds between cachexia and sarcopenic obesity should be investigated. Despite these considerations, this is the first assemblage of available literature comparing objective physical function between cancer patients with and without cachexia and changes in objective physical function from interventional studies utilizing cancer cachexia cohorts.

## 6. Conclusions and Future Directions

The most frequently measured physical function assessment in both cross-sectional and interventional studies was HGS. This is consistent with a recent systematic review, which reported the frequency and diversity of physical function outcomes in cancer cachexia trials involving adult participants with *n* > 40 and duration ≥14 days [16]. That review reported the proportion of trials that observed statistically significant effects on each physical function outcome but did not report magnitudes of effect. Guidelines for physical function assessment and tracking in cancer cachexia are still lacking standardization and clinical relevance. Even HGS, despite being the most widely utilized functional assessment here, was not consistently measured, nor was methodology consistently or clearly reported. No studies reviewed here derived their own MID, MIC, and/or MCID. Many utilized previously published criteria, often from cohorts without similar disease/treatment burden, for classifying the clinical relevance of their own observations. However, MCIDs should not be generalized too broadly due to disease/treatment-specific impacts on outcomes of interest and/or variability in MCID derivation. 

The current data suggest that patients with cachexia may display clinically important HGS impairment, which may be moderately improved by interventions aiming to increase muscle mass. This suggests that close monitoring of HGS may be suitable for tracking treatment response and/or guiding adjustments during the clinical management of cancer cachexia. Whether clinical trials should incorporate HGS into entry criteria as a measure of functional impairment and/or whether they should monitor HGS as an indicator of cachexia progression and/or intervention success cannot be determined by the small number of cohorts reviewed here. Although HGS was the most common assessment reported, it was not impacted consistently, particularly regarding statistical significance. This may be because it is not a sensitive measure of physical function in all cancer cachexia settings or perhaps, in the studies where HGS was not impacted, this outcome was not physiologically relevant and/or they were underpowered or not well controlled for detecting functional differences/changes. Future studies should focus on deriving cohort-specific clinically relevant functional impairment and functional change criteria using multiple outcome measures that assess varying aspects of physical function. This will require the incorporation of correlation and receiver operating characteristic analyses to determine suitable patient-reported anchors for physical function outcomes. As research on cancer cachexia continues, reviews should be consistently updated with the growing knowledge of pathophysiology and clinical management, especially regarding factors related to physical function, quality of life, and survival.

## Figures and Tables

**Table 1:** Cross-sectional Studies Comparing Objective Physical Function between Cancer Patients with and without Cachexia.

	Study Design and Outcomes	Outcome Averages by Group ^A^	MID Estimates ^B^
Anderson et al., 2021 [34]	**Cohort:** Solid tumors (mostly gastrointestinal and genitourinary) **Cachexia:** Fearon et al., 2011 [5] **Arms**: CC (48M), CNC (48M); also, non-cancer control, not included here **Objective Function:** HGS (mean of max of both hands), SCP, and 1-RM ^C^ **Subjective Function:** KPS, ECOG, and FACIT-F **PR-QOL:** ASAS and FACIT-F ^D^ **1°:** Functional performance ○ROC analyses were performed to identify objective function criteria for characterizing cachexia	**Objective Physical Function**	**Objective Physical Function**
		* SCP (W): 280 (CC) vs. 430 (CNC); −150	**SCP:** 68.2 (ROC: 336W, AUC = 0.80)
		**HGS (kg):** 34 (CC) vs. 37 (CNC); −3	**HGS:** 3.2
		* ChPr (kg): 29 (CC) vs. 45 (CNC); −16	**ChPr:** 5.4 (ROC: 38.5 kg, AUC = 0.75)
		**UpBa (kg):** 40 (CC) vs. 52 (CNC); −12	**UpBa:** 4.6 (ROC: 46 kg, AUC = 0.72)
		**LaPu (kg):** 40 (CC) vs. 51 (CNC); −11	**LaPu:** 5.0 (ROC: 46.5 kg, AUC = 0.71)
		**KnFl (kg):** 50 (CC) vs. 60 (CNC); −10	**KnFl:** 6.3
		**KnEx (kg):** 51 (CC) vs. 65 (CNC); −14	**KnEx:** 6.1
		**HipEx (kg):** 28 (CC) vs. 25 (CNC); +3	**HipEx:** 3.9
	
		**Patient-Reported Outcomes**	**Patient-Reported Outcomes**
		* ECOG: 1.0 (CC) vs. 0.0 (CNC); +1.0	**ECOG:** 0.2
		**KPS:** 90 (CC) vs. 100 (CNC); −10	**KPS:** 2.3
		**FACIT Fatigue:** 37.5 (CC) vs. 38.0 (CNC); −0.5	**FACIT Fatigue:** 4.1
		**FACIT Function:** 18.3 (CC) vs. 21.0 (CNC); −2.7	**FACIT Function:** 2.4
		**FACIT-F Total:** 107.3 (CC) vs. 118.3 (CNC); −11	**FACIT-F Total:** 8.0
		**ASAS Total:** 74.5 (CC) vs. 81.0 (CNC); −6.5	**ASAS Total:** 5.7
Burney et al., 2012 [35]	**Cohort:** Various tumor types **Cachexia:** WL > 5% in prior 6 mos **Arms**: CC (45M), CNC (50M); also, non-cancer control, not included here **Objective Function:** HGS (sum of both hands), TPPT, and GGT **Subjective Function:** KPS and ECOG **PR-QOL:** ASAS and FACIT-F **1°:** Relationship between testosterone, inflammation, and symptom burden	**Objective Physical Function**	**Objective Physical Function**
		**HGS (kg):** n/a (sum of both hands)	**HGS:** n/a
		**TPPT (s):** 37 (CC) vs. 38 (CNC); −1	**TPPT:** 0.6
		**GGT (s):** 8.2 (CC) vs. 8.5 (CNC); −0.3	**GGT:** 0.13
	
		**Patient-Reported Outcomes**	**Patient-Reported Outcomes**
		**KPS:** 87 (CC) vs. 89 (CNC); −2	**KPS:** 3.3
		**ECOG:** 0.8 (CC) vs. 0.9 (CNC); −0.1	**ECOG:** 0.2
		**FACIT F-Total:** 103.7 (CC) vs. 107.0 (CNC); −3.3	**FACIT-F Total:** 11.3
		**ASAS Total:** 70 (CC) vs. 62.6 (CNC); +7.4	**ASAS Total:** 5.9
Cong et al., 2022 [36]	**Cohort:** Various tumor types **Cachexia:** WL > 5% in 12 mos and ≥3: low strength, fatigue, anorexia, low muscle, and abnormal labs **Arms**: CC (351) and CNC (3380) sex unreported **Sex-/Age-matched**: CC (347) and CNC (347) **Objective Function:** HGS (undefined) **Subjective Function:** KPS and PG-SGA **PR-QOL:** PG-SGA **1°:** PG-SGA prediction of cachexia (ROC)	**Objective Physical Function**	**Objective Physical Function**
		* HGS (kg): 18.8 (CC) vs. 24.8 (CNC); −6	**HGS:** 3.2
		○* Matched groups: 18.9 (CC) vs. 23.9 (CNC); −5	Matched: 2.8
	
		**Patient-Reported Outcomes**	**Patient-Reported Outcomes**
		* KPS: 79.2 (CC) vs. 88.9 (CNC); −9.7	**KPS:** 3.4
		○* Matched groups: 79.6 (CC) vs. 87.1 (CNC); −7.5	Matched: 3.4
		* PG-SGA: 10.9 (CC) vs. 4.7 (CNC); +6.2	**PG-SGA:** 1.2
		○* Matched groups: 10.8 (CC) vs. 6.2 (CNC); +4.6	Matched: 1.3 (ROC: 6.5; AUC = 0.85)
Dolin et al., 2023 [37]	**Cohort:** Colorectal cancer **Cachexia:** Fearon et al., 2011 [5] **Arms**: CC (7M/16F) and CNC (5M/36F) **Objective Function:** HGS (max of dominant hand), 5STS, normal gait speed, and 6MWT **Subjective Function and PR-QOL:** n/a **1°:** Preoperative sarcopenia and cachexia	**Objective Physical Function**	**Objective Physical Function**
		**HGS (kg):** M: 41.0 (CC) vs. 34.6 (CNC); +6.4 F: 21.7 (CC) vs. 21.0 (CNC); +0.7	**HGS:** only HGS reported by gender; M: 3.6, F: 1.6
		**5STS (s):** 10.0 (CC) vs. 10.4 (CNC); −0.4 [median reported for 5STS]	**5STS:** unable to derive
		**Gait (m/s):** 1.1 (CC) vs. 1.0 (CNC); +0.01	**Gait:** 0.10
		**6MWT (m):** 390 (CC) vs. 349 (CNC); +41	**6MWT:** 44
Hadzibegovic et al., 2023 [38]	**Cohort:** Mostly advanced solid tumors **Cachexia:** Fearon et al., 2011 [5] **Arms**: CC (70M/78F), CNC (93M/92F), and non-cancer control, not included here **Objective Function:** HGS (max of either hand), normal gait speed, and 6MWT **Subjective Function:** KPS and ECOG **PR-QOL:** EQ-5D-5L **1°:** HGS	**Objective Physical Function**	**Objective Physical Function**
		* HGS (kg): 28.3 (CC) vs. 33.6 (CNC); −5.3	**HGS:** 4.1
		* Gait (m/s): 1.0 (CC) vs. 1.2 (CNC); −0.2	**Gait:** 0.13
		**6MWT (m):** 419 (CC) vs. 450 (CNC); −31	**6MWT:** 31.3
	
		**Patient-Reported Outcomes**	**Patient-Reported Outcomes**
		* ECOG: 2.1 (CC) vs. 1.4 (CNC); +0.7	**ECOG:** 0.4
		* KPS: 65 (CC) vs. 79 (CNC); −14	**KPS:** 7.3
		* EQ-5D-5L: 0.66 (CC) vs. 0.73 (CNC); −0.07	**EQ-5D-5L:** 0.09
Ohmae et al., 2023 [39]	**Cohort:** Recent head and neck diagnosis **Cachexia:** Fearon et al., 2011 [5] **Arms**: CC (16M/7F) and CNC (35M/6F) **Objective Function:** HGS (max of either hand), IKEF, gait speed (normal and max), 5STS, steps/d, and activity time **Subjective Function and PR-QOL:** n/a **1°:** Muscle mass and quality, physical function, and activity	**Objective Physical Function**	**Objective Physical Function**
		* HGS (kg): 26.7 (CC) vs. 33.2 (CNC); −6.6	**HGS:** 1.9
		* IKEF (%BW): 44.5 (CC) vs. 58.2 (CNC); −13.7	**IKEF:** 2.8
		* Gait (m/s): 1.04 (CC) vs. 1.21 (CNC); −0.17	**Gait:** 0.06
		* **Gait-Max (m/s):** 1.56 (CC) vs. 1.77 (CNC); −0.21	**Gait-max:** 0.07
		* 5STS (s): 10 (CC) vs. 8.1 (CNC); +1.9	**5STS:** 0.53
		**Steps/d:** 2975 (CC) vs. 3210 (CNC); −235	**Steps/d:** 601
		**Activity time (mins/d):**	**Activity time:**
		○**Total:** 828 (CC) vs. 829 (CNC); −1	○**Total:** 58
		○**<3 METs:** 807 (CC) vs. 781 (CNC); +26	○**<3 METs:** 47.2
		○* >3 METs: 19 (CC) vs. 29 (CNC); −10	○**≥3 METs:** 4.7
Stephens et al., 2012 [40]	**Cohort:** Gastrointestinal cancer at any stage **Cachexia:** WL ≥ 10% pre-morbid weight **Arms**: CC (15M/9F), CNC (20M/10F); also, non-cancer control, not included here **Objective Function:** HGS, IKEF, and LLEP **Subjective Function:** KPS and EORTC QLQ-C30 physical function **PR-QOL:** EORTC QLQ-C30 fatigue **1°:** Relationship between cachexia, QOL, muscle mass, and function	**Objective Physical Function**	**Objective Physical Function**
		**HGS (kg):** M: 38 (CC) vs. 38 (CNC); 0	**HGS (kg):** M: 3.3, F: 3.3
		F: 22 (CC) vs. 27 (CNC); −5	
		**IKEF (N):** M: 243 (CC) vs. 288 (CNC); −45	**IKEF (N):** M: 30, F: 23.3
		* F: 159 (CC) vs. 252 (CNC); −93	
		**IKEF (N/kg):** M: 3.2 (CC) vs. 3.7 (CNC); −0.5	**IKEF (N/kg):** M: 0.4, F: 0.3
		F: 2.7 (CC) vs. 3.9 (CNC); −1.2	
		**LLEP (W):** M: 98 (CC) vs. 123 (CNC); −25	**LLEP (W):** M: 19.3, F: 4.7
		F: 59 (CC) vs. 63 (CNC); −4	
		**LLEP (W/kg):** M: 1.3 (CC) vs. 1.5 (CNC); −0.2	**LLEP (W/kg):** M: 0.17, F: 0.07
		F: 1.0 (CC) vs. 1.0 (CNC); 0	
	
		**Patient-Reported Outcomes**	**Patient-Reported Outcomes**
		**KPS:** M: 79 (CC) vs. 84 (CNC); −5	**KPS:** M: 3.7, F: 4
		* F: 77 (CC) vs. 90 (CNC); −13	
		**QLQ-C30 Function:** M: 60 (CC) vs. 83 (CNC); −23	**QLQ-C30 Function:** M: 7, F: 8
		F: 73 (CC) vs. 86 (CNC); −13	
		**QLQ-C30 Fatigue:** M: 56 (CC) vs. 27 (CNC); +29	**QLQ-C30 Fatigue:** M: 7.7, F: 11.3
		F: 44 (CC) vs. 26 (CNC); +18	

* Significant (*p* ≤ 0.05) between-group difference as reported by individual studies. ^A^ Group means are displayed as reported by individual studies with differences in means [cancer cachexia (CC) − cancer no cachexia (CNC)] displayed after the semicolon [CC − CNC]. ^B^ Minimal important difference (MID) was derived by dividing CNC group standard deviation (SD) by three (distribution-based Equation (2)); SD was calculated using the sample size and standard error of the mean, if available when SD was not. ^C^ One-repetition maximum (1-RM) strength: Chest Press (ChPr), Upper Back Seated Row (UpBa), Latissimus Pull-Down (LaPu), Knee Extension/Flexion (KnEx/KnFl), Hip Extension (HipEx). ^D^ Unadjusted scores are reported, despite adjusted scores being reported in the original paper, for comparison to the non-cancer control. Abbreviations: **1°**, primary outcome; WL, weight loss; mos, months; M, male; F, female; HGS, hand grip strength; TPPT, timed physical performance test; GGT, get-up-and-go test; KPS, Karnofsky Performance Score; ECOG, Eastern Cooperative Oncology Group; FACIT-F, Functional Assessment of Chronic Illness Therapy-Fatigue; PR-QOL, patient-reported quality of life; ASAS, Anderson Symptom Assessment Score; ROC, receiver operating characteristic; AUC, area under the curve; IKEF, isometric knee extension force; LLEP, lower limb extensor power; EORTC QLQ-C30, European Organization for the Research and Treatment of Cancer Quality of Life Questionnaire; PG-SGA, Patient-Generated Subjective Global Assessment; SCP, stair climb power; W, watts; kg, kilograms; m/s, meters/second; 5STS, five times sit-to-stand; METs, metabolic equivalents; 6MWT, 6-minute walk test; EQ-5D-5L; EuroQol-5Dimensions-5Levels.

**Table 2:** Interventional Studies Comparing Objective Functional Changes in Patients with Cancer Cachexia.

	Cohort and Design	Within-Group Mean Changes ^A^	MIC Estimates ^B^
**Muscle-Targeting Anabolic Interventions**			
Dobs et al., 2013 [43]	**Cohort:** Various cancer types **Cachexia:** ≥2% WL in prior 6 mos **Arms:** EXP: 1 mg/d (16M/16F) or 3 mg/d (21M/13F) enobosarm, CON: placebo (21M/13F) ●16 wks; **1°:** Lean body mass	**Objective Physical Function** (median change)	**Objective Physical Function**
		**HGS (kg):** 1 mg: 2.0, 3 mg: 0.0, CON: 0.1; + 1.9 (1 mg), −0.1 (3 mg)	**HGS:** MIC-2 (0.3)
		**SCP (W):** * 1 mg: 19.9, * 3 mg: 12.8, CON: 11.3; +8.6 (1 mg), +1.5 (3 mg)	**SCP:** MIC-2 (13.1)
		**Gait-habitual (m/s):** 1 mg: −0.40, 3 mg: 0, CON: −0.04; −0.36 (1 mg), +0.04 (3 mg)	**Gait:** MIC-2 (0.53)
	
		**Patient-Reported Outcomes** (mean change)	**Patient-Reported Outcomes**
		**FAACT Total:** * 1 mg: 9.5, 3 mg: 4.1, CON: 2.3; +7.2 (1 mg), +1.8 (3 mg)	**FAACT Total:** MIC-2 (5.5)
		**FACIT-F Total:** * 1 mg: 9.4, 3 mg: 1.0, CON: 1.6; +7.8 (1 mg). −0.6 (3 mg)	**FACIT-F Total:** MIC-2 (5.9)
		**FAACT-Anorexia/Cachexia:** * 1 mg: 7.0, 3 mg: 3.1, CON: 2.3; +4.7 (1 mg), +0.8 (3 mg)	**FAACT-Anorexia/Cachexia:** MIC-2 (3.2)
	
			MIC-1 n/a (SD or IRQ NR)
Solheim et al., 2017 [44] Pre-MENAC Study	**Cohort:** Stage III/IV NSCLC or inoperable pancreatic **Cachexia:** <20% WL in prior 6 mos (~50% per arm had >5% WL in 6 mos) **Arms:** EXP: 300 mg/d celecoxib + oral nutritional supplement + nutrition counseling + exercise (15M/10F), CON: usual care (11M/10F) ●6 wks; **1°:** Feasibility	**Objective Physical Function**	**Objective Physical Function**
		**HGS (kg)**: EXP: −0.4, CON: −0.7; +0.3	**HGS:** MIC-1 (4.2), MIC-2 (1.7)
		**6MWT (m)**: EXP: 0.1, CON: 20.3; −20.2	**6MWT:** MIC-1 (29.1), MIC-2 (18.0)
		**Activity (steps/d):** EXP: −536, CON: 981; −1517	**Activity:** MIC-1 (870), MIC-2 (564)
	
		**Patient-Reported Outcomes**	**Patient-Reported Outcomes**
		**PG-SGA**: EXP: −0.8, CON: 0.1; −0.9	**PG-SGA:** MIC-1 (2.1), MIC-2 (2.2)
		**Fatigue Severity Scale**: EXP: 0.7, CON: 0.2; +0.5	**Fatigue:** MIC-1 (0.5), MIC-2 (0.6)
Wright et al., 2018 [45]	**Cohort:** Cervical or head and neck **Cachexia:** ≥5% WL in prior 12 mos **Arms:** EXP: 100 mg/d testosterone enanthate (3M/6F), CON: placebo (7M/5F) ●7 wks; **1°:** Lean body mass	**Objective Physical Function** **Leg torque (units undefined):** EXP: 6.3%, CON: 2.9%; +3.4 **Leg power (units undefined):** EXP: 7.0%, CON: 3.8%; +3.2 **SPPB Total:** EXP: 1.4, CON: 0.3; +1.1 **Patient-Reported Outcomes** **FACT-G Function:** EXP: 1.2, CON: −2.0; +3.2 **FACT-G Total:** EXP: 4.5, CON: −3.1; +7.6	MIC-1 and -2 n/a (SD or IRQ NR)
Maccio et al., 2012 [46]	**Cohort:** Gynecological cancer **Cachexia:** ≥5% WL in prior 3 mos **Arms:** EXP: 4 g/d carnitine + 300 mg/d celecoxib + 600 mg/d lipoic acid + 2.7 g/d carbocysteine + 320 mg/d megestrol acetate (61F), CON: 320 mg/d megestrol acetate (63F) ●16 wks; **1°:** Lean body mass, resting energy expenditure, fatigue, and QOL	**Objective Physical Function**	**Objective Physical Function**
		**HGS (kg):** EXP: 3.0, CON: −1.1; +4.1	**HGS:** MIC-1 (2.7)
	
		**Patient-Reported Outcomes**	**Patient-Reported Outcomes**
		**ECOG:** * EXP: −0.6, * CON: −0.5; −0.1	**ECOG:** MIC-1 (0.3)
		**#Fatigue (MFSI-SF):** EXP: −6.4, CON: 0.9; −7.3	**Fatigue:** MIC-1 (5.3)
		**#EORTC QLQ-C30:** * EXP: 7.5, CON: 4.1; +3.4	**EORTC QLQ-C30:** MIC-1 (4.3)
	
			MIC-2 n/a (SD or IRQ NR)
Cereda et al., 2019 [47]	**Cohort:** Advanced cancer **Cachexia:** ≥10% WL in prior 6 mos **Arms:** EXP: nutrition counseling + whey protein (35F/47M), CON: nutrition counseling (31F/53M) ●3 mos; **1°:** Phase angle	**Objective Physical Function**	**Objective Physical Function**
		**#HGS (kg):** EXP: 1.4, CON: −0.9; +2.3	**HGS:** MIC-1 (2.9), MIC-2 (1.5)
	
		**Patient-Reported Outcomes**	**Patient-Reported Outcomes**
		**EORTC QLQ-C30 Global QOL:** EXP: 2.9, CON: 0.5; +2.4	**EORTC QLQ-C30 Global QOL:** MIC-1 (6.8), MIC-2 (5.5)
Jatoi et al., 2017 [48]	**Cohort:** Incurable malignancy **Cachexia:** ≥5 pounds WL in prior 2 mos **Arms:** EXP: creatine 20 g/d for 5 days then 2 g/d (51F/83M), CON: placebo (49F/80M) ●Median duration: EXP: 54.5 days, CON: 64 days; **1°:** Weight	**Objective Physical Function**	**Objective Physical Function**
		**HGS (kg):** EXP: −0.2, CON: −0.8; +0.6	**HGS:** MIC-1 (3.3), MIC-2 (2.4)
**Appetite Stimulants**			
Currow et al., 2017 [11] ROMANA3	**Cohort:** Unresectable III/IV NSCLC **Cachexia:** WL > 5% in prior 6 mos or BMI < 20 kg/m^2^ **Arms:** EXP: 100 mg/d anamorelin (262M/83F), CON: placebo (125M/43F) ●ROMANA ½ plus 12 wks (24 wks total); **1°:** Safety/Tolerability	**Objective Physical Function**	**Objective Physical Function**
		**HGS (kg):** EXP: −0.8, CON: −0.6; −0.2	**HGS:** MIC-1 (3.8), MIC-2 (0.3)
	
		**Patient-Reported Outcomes**	**Patient-Reported Outcomes**
		**FAACT-Anorexia/Cachexia:** EXP: 4.5, CON: 3.2; +1.3	**FAACT-Anorexia/Cachexia:** MIC-1 (2.8), MIC-2 (0.3)
Madeddu et al., 2012 [12]	**Cohort:** Advanced cancer of any site **Cachexia:** ≥5% WL in prior 6 mos **Arms:** EXP: 4 g/d carnitine + 300 mg/d celecoxib + 320 mg/d megestrol acetate (16M/11F), CON: carnitine + celecoxib (17M/12F) ●4 mos; **1°:** Lean body mass and activity	**Objective Physical Function** **HGS-D (kg):** EXP: 1.7, CON: 3.8; −2.1 6MWT (m): * EXP: 53, * CON: 45; +8 **Activity (steps/d):** EXP: 1328, CON: 390; +938 **Patient-Reported Outcomes** **ECOG:** * EXP: −0.3, * CON: −0.4; +0.1 **Fatigue (MFSI-SF):** * EXP: −8.8, * CON: −7.4; −1.4	MIC-1 and -2 n/a (SD or IRQ NR)
Temel et al., 2016 [14] ROMANA 1 and 2	**Cohort:** Unresectable III/IV NSCLC **Cachexia:** WL ≥5% in prior 6 mos or BMI < 20 kg/m^2^ **Arms:** EXP: 100 mg/d anamorelin, CON: placebo ●ROMANA 1 “R1” (EXP: 247M/76F, CON: 121M/40F) and 2 “R2” (EXP: 240M/90F, CON: 122M/43F) ●12 wks; **1°:** Lean body mass and HGS	**Objective Physical Function**	**Objective Physical Function**
		**HGS-ND (kg):** median change	**HGS-ND:**
		●R1: EXP: −1.1, CON: −1.6; +0.5	●R1: MIC-1 (4.7), MIC-2 (0.5)
		●R2: EXP: −1.6, CON: −1.0; −0.6	●R2: MIC-1 (4.1), MIC-2 (0.4)
	
		**Patient-Reported Outcomes**	**Patient-Reported Outcomes**
		**FACIT-Fatigue:**	**FACIT-Fatigue:**
		●R1: EXP: 0.3, CON: −1.9; +2.2	●R1: MIC-1 (3.6), MIC-2 (0.5)
		●R2: EXP: 1.4, CON: 1.2; +0.2	●R2: MIC-1 (3.6), MIC-2 (0.4)
		**FAACT-Anorexia/Cachexia:**	**FAACT-Anorexia/Cachexia:**
		●#R1: EXP: 4.1, CON: 1.9; +2.2	●R1: MIC-1 (2.9), MIC-2 (0.3)
		●#R2: EXP: 3.5, CON: 1.3; +2.2	●R2: MIC-1 (2.9), MIC-2 (0.3)
Garcia et al., 2015 [15] ^C^	**Cohort:** Incurable malignancy **Cachexia:** WL ≥5% in prior 6 mos **Arms:** EXP: 50 mg/d anamorelin (28M/16F), CON: placebo (23M/15F) ●12 wks; **1°:** Lean body mass	**Objective Physical Function**	**Objective Physical Function**
		**HGS-ND (kg):** EXP: 1.6, CON: 0.7; +0.9	**HGS-ND:** MIC-1 (3.6), MIC-2 (1.9)
	
		**Patient-Reported Outcomes**	**Patient-Reported Outcomes**
		**ASAS Fatigue:** EXP: 0.6, CON: −0.4; +1	**ASAS Fatigue:** MIC-1 (0.9), MIC-2 (1.2)
		**#ASAS Total:** EXP: 3.6, CON: 0.06; +3.5	**ASAS Total:** MIC-1 (5.7), MIC-2 (6.7)
Herodes et al., 2023 [49] ^C^	**Cohort:** Active malignancy **Cachexia:** WL ≥5% in prior 6 mos, ≥10% in prior 12 mos, or ≥2% in prior 6 mos with BMI < 20 kg/m^2^ **Arms:** EXP: 0.25 – 0.5 mg/kg/d macimorelin (10M), CON: placebo (4M/1F) ●7 days; **1°:** Weight, insulin-like growth factor-1, QOL, and safety ●HGS was reported as an average of both hands	**Objective Physical Function** (medians)	**Objective Physical Function**
		**HGS (kg):** EXP: −1.3, CON: 1.4; −2.7	**HGS:** MIC-1 (2.3), MIC-2 (0.3)
		**SCP (W):** EXP: −5.1, CON: −5.9; +0.8	**SCP:** MIC-1 (11.4), MIC-2 (3.5)
	
		**Patient-Reported Outcomes** (medians)	**Patient-Reported Outcomes**
		**ECOG:** EXP: 0, CON: 0; 0	**ECOG:** MIC-1 (0.2), MIC-2 (0)
		**KPS:** EXP: 0, CON: 0; 0	**KPS:** MIC-1 (1.8), MIC-2 (0)
		**FACIT-Fatigue:** EXP: −1.0, CON: 3.0; −4	**FACIT-Fatigue:** MIC-1 (2.1), MIC-2 (2.0)
		**FACT-G Function:** EXP: 1.0, CON: −0.5; +1.5	**FACT-G Function:** MIC-1 (1.4), MIC-2 (0.7)
Kouchaki et al., 2018 [50]	**Cohort:** GI cancer **Cachexia:** ≥5% WL in prior 6 mos or BMI < 20 kg/m^2^ **Arms:** EXP: 320 mg/d megestrol acetate + 200 mg/d celecoxib (17F/28M), CON: megestrol acetate (17F/28M) ●2 mos; **1°:** Weight	**Objective Physical Function**	**Objective Physical Function**
		**HGS (kg):** EXP: 3.9, * CON: 6.8; −2.9	**HGS:** MIC-1 (2.8)
	
		**Patient-Reported Outcomes**	**Patient-Reported Outcomes**
		**ECOG:** EXP: −0.6, * CON: −0.8; +0.2	**ECOG:** MIC-1 (0.3)
		**EORTC QLQ-C30:** * EXP: 15.7, * CON: 19.8; −4.1	**EORTC QLQ-C30:** MIC-1 (4.5)
	
			MIC-2 n/a (SD or IRQ NR)
Wen et al., 2012 [51] ^D^	**Cohort:** Advanced cancer of any site **Cachexia:** WL > 5% in prior 3 mos **Arms:** EXP: 320 mg/d megestrol acetate + 100 mg/d thalidomide (28M/18F), CON: megestrol acetate (27M/20F) ● 2 mos; **1°:** Weight, fatigue, and QOL	**Objective Physical Function**	**Objective Physical Function**
		**#HGS (kg):** * EXP: 1.1, CON: 0.6; +0.5	**HGS:** MIC-1 (3.9), MIC-2 (0.4)
	
		**Patient-Reported Outcomes**	**Patient-Reported Outcomes**
		**#ECOG:** * EXP: −0.4, CON: −0.1; −0.3	**ECOG:** MIC-1 (0.2), MIC-2 (0.1)
		**#Fatigue (MFSI-SF):** * EXP: −2.6, CON: 0.2; −2.8	**Fatigue:** MIC-1 (7.2), MIC-2 (1.8)
Hunter et al., 2021 [52]	**Cohort:** Solid tumors **Cachexia:** Fearon et al., 2011 [5] **Arms:** EXP: 15 mg/d mirtazapine (26F/34M), CON: placebo (30F/30M) ●28 days; **1°:** Appetite	**Objective Physical Function**	**Objective Physical Function**
		**HGS (kg):** EXP: −0.8, CON: 0; −0.8	**HGS:** MIC-1 (2.1), MIC-2 (0.2)
	
		**Patient-Reported Outcomes**	**Patient-Reported Outcomes**
		**FAACT-Total:** EXP: 2.2, CON: 0.6; +1.6	**FAACT-Total:** MIC-1 (5.7), MIC-2 (3.6)
**Immunomodulators and Oral Supplements**			
Laviano et al., 2020 [53]	**Cohort:** NSCLC starting chemotherapy **Cachexia:** Various WL/BMI ranges **Arms:** EXP: oral nutritional supplement (9F/17M, 38.5% CC), CON: isocaloric match (8F/21M, 48.3% CC) ●12 wks; **1°:** Safety and tolerability ●%CC reported by Fearon criteria [5]	**Objective Physical Function**	**Objective Physical Function**
		**HGS-D (kg):** EXP: 0.2, CON: −3.1; +3.3	**HGS-D:** MIC-2 (4.3)
		**HGS-ND (kg):** EXP: 0.7, CON: −1.6; +2.3	**HGS-ND:** MIC-2 (1.5)
		**Activity (steps/d):** EXP: 647, CON: −202; +849	**Activity:** MIC-2 (768)
	
			MIC-1 n/a (SD or IRQ NR)
Wiedenmann et al., 2008 [54]	**Cohort:** Pancreatic cancer **Cachexia:** WL ≥10% of pre-morbid weight or ≥5% in the prior 90 days **Arms:** EXP: 3 mg/kg/d (13M/17F) or 5 mg/kg/d (15M/14F) infliximab, CON: placebo (20M/10F) ●8 wks; **1°:** Lean body mass	**Objective Physical Function**	**Objective Physical Function**
		**6MWT (m):** 3 mg: −157.5, 5 mg: −87.8, CON: −114.1; −43.4 (3 mg), +26.3 (5 mg)	**6MWT:** MIC-2 (62.3)
	
		**Patient-Reported Outcomes**	**Patient-Reported Outcomes**
		**FACIT-F Total:** 3 mg: −3.7, 5 mg: 2.3, CON: −3.5; −0.2 (3 mg), +5.8 (5 mg)	**FACIT-F Total:** MIC-2 (2.8)
		**FAACT Total:** 3 mg: −0.8, 5 mg: 3.4, CON: 0.2; −1 (3 mg), +3.2 (5 mg)	**FAACT Total:** MIC-2 (3.3)
		**SF-36 Physical Function:** 3 mg: −3.4, 5 mg: 0.5, CON: 0.1; −3.5 (3 mg), +0.4 (5 mg)	**SF-36 Physical Function:** MIC-2 (2.2)
	
			MIC-1 n/a (SD or IRQ NR)
Famil-Dardashti et al., 2020 [55]	**Cohort:** Advanced solid tumors **Cachexia:** ≥5% WL in prior 2 mos **Arms:** EXP: herbal supplements (9F/16M), CON: placebo (9F/13M) ●2 mos; **1°:** Weight gain	**Objective Physical Function**	**Objective Physical Function**
		**#HGS (kg):** EXP: 2.4, CON: −0.5; +2.9	**HGS:** MIC-1 and -2 n/a (SD or IRQ NR)
	
		**Patient-Reported Outcomes**	**Patient-Reported Outcomes**
		**ESAS Fatigue:** EXP: −0.1, CON: 0.2; −0.3	**ESAS Fatigue:** MIC-1 (0.1), MIC-2 (0.03)
Xie et al., 2018 [56]	**Cohort:** Stage IV SCLC **Cachexia:** Fearon et al., 2011 [5] **Arms:** EXP: 150 mg/d thalidomide and 2700 mg/d cinobufagin (5F/22M), CON: cinobufagin (7F/20M) ●2 mos; **1°:** Weight gain	**Objective Physical Function**	**Objective Physical Function**
		**#HGS (kg):** EXP: 0.9, CON: −0.2; +1.1	**HGS:** MIC-1 (0.9), MIC-2 (0.3)
	
		**Patient-Reported Outcomes**	**Patient-Reported Outcomes**
		**#EORTC QLQ-C30:** EXP: −8.1, CON: −0.5; −7.6	**EORTC QLQ-C30:** MIC-1 (1.8), MIC-2 (1.0)
Gordon et al., 2005 [57]	**Cohort:** Inoperable pancreatic cancer **Cachexia:** >10% WL in prior 6 mos **Arms:** EXP: 200 mg/d thalidomide (13M/11F), CON: placebo (12M/11F) ●24 wks (HGS assessed at 8 wks); **1°:** Weight	**Objective Physical Function** **HGS-ND (kg):** EXP: −2.5, CON: −1.0; −1.5	MIC-1 and -2 n/a (SD or IRQ NR)

Statistically significant (*p* ≤ 0.05) * within-group difference or change or # between-group difference, as reported by individual studies. ^A^ Within-group mean (standard deviation, SD) changes [endpoint–baseline] are displayed as reported by individual studies, unless otherwise noted, with the between-group difference in change displayed after the semicolon [experimental (EXP)–control/placebo (CON)]. ^B^ The minimal important change (MIC) for outcomes was calculated by dividing CON baseline SD (MIC-1) or SD of change score (MIC-2) by three (distribution-based Equation 2.1); SD was calculated by dividing the interquartile range (IQR) by 1.35 or by multiplying the standard error of the mean (SEM) with the square root of the sample size, if available, instead of SD. ^C^ The SD used to calculate MID was obtained from the original data provided by co-author JM Garcia as it was not reported in the original paper. ^D^ Change was reported in the original publication as baseline–endpoint; so, the signs of change were flipped here to represent endpoint–baseline. Abbreviations: **1°**, primary outcome; WL, weight loss; mos, months; M, male; F, female; d, day; wk/s, week/s; kg, kilograms; m/s, meters/second; HGS, hand grip strength (N/D, Non/Dominant); A/ESAS, Anderson/Edmonton Symptom Assessment Scale; NSCLC, Non-Small Cell Lung Cancer; BMI, body mass index; QOL, quality of life; SCP, stair climb power; W, watts; ECOG, Eastern Cooperative Oncology Group; KPS, Karnofsky Performance Score; FACIT-F, Functional Assessment of Chronic Illness Therapy-Fatigue; FAACT, Functional Assessment of Anorexia/Cachexia Therapy Score; GI, gastrointestinal; PG-SGA, Patient-Generated Subjective Global Assessment; MFSI-SF, Multidimensional Fatigue Symptom Inventory-Short Form; SF-36, Short form-36; SPPB, Short Physical Performance Battery; FACT-G, Functional Assessment of Cancer Therapy-General; EORTC QLQ-C30, European Organization for the Research and Treatment of Cancer Quality of Life Questionnaire.

## References

[1] Amano K., Arakawa S., Hopkinson J.B., Baracos V.E., Oyamada S., Koshimoto S., Mori N., Ishiki H., Morita T., Takeuchi T. (2023). Factors Associated with Practice of Multimodal Care for Cancer Cachexia Among Physicians and Nurses Engaging in Cancer Care. JCO Oncol. Pract..

[2] Gouldthorpe C., Power J., Taylor A., Davies A. (2023). Specialist Palliative Care for Patients with Cancer: More Than End-of-Life Care. Cancers.

[3] Ferrell B.R., Temel J.S., Temin S., Smith T.J. (2017). Integration of Palliative Care Into Standard Oncology Care: ASCO Clinical Practice Guideline Update Summary. J. Oncol. Pract..

[4] Vagnildhaug O.M., Balstad T.R., Almberg S.S., Brunelli C., Knudsen A.K., Kaasa S., Thronaes M., Laird B., Solheim T.S. (2018). A cross-sectional study examining the prevalence of cachexia and areas of unmet need in patients with cancer. Support. Care Cancer.

[5] Fearon K., Strasser F., Anker S.D., Bosaeus I., Bruera E., Fainsinger R.L., Jatoi A., Loprinzi C., MacDonald N., Mantovani G. (2011). Definition and classification of cancer cachexia: An international consensus. Lancet Oncol..

[6] Evans W.J., Morley J.E., Argiles J., Bales C., Baracos V., Guttridge D., Jatoi A., Kalantar-Zadeh K., Lochs H., Mantovani G. (2008). Cachexia: A new definition. Clin. Nutr..

[7] Rantanen T., Harris T., Leveille S.G., Visser M., Foley D., Masaki K., Guralnik J.M. (2000). Muscle strength and body mass index as long-term predictors of mortality in initially healthy men. J. Gerontol. A Biol. Sci. Med. Sci..

[8] Gale C.R., Martyn C.N., Cooper C., Sayer A.A. (2007). Grip strength, body composition, and mortality. Int. J. Epidemiol..

[9] Fram J., Vail C., Roy I. (2022). Assessment of Cancer-Associated Cachexia—How to Approach Physical Function Evaluation. Curr. Oncol. Rep..

[10] Crawford J., Johnston M., Hancock M., Small S., Taylor R., Dalton J., Steiner M. (2014). Enobosarm, a selective androgen receptor modulator (SARM) increases lean body mass (LBM) in advanced NSCLC patients: Updated results of two pivotal, international phase 3 trials. Support. Care Cancer.

[11] Currow D., Temel J.S., Abernethy A., Milanowski J., Friend J., Fearon K.C. (2017). ROMANA 3: A phase 3 safety extension study of anamorelin in advanced non-small-cell lung cancer (NSCLC) patients with cachexia. Ann. Oncol..

[12] Madeddu C., Dessi M., Panzone F., Serpe R., Antoni G., Cau M.C., Montaldo L., Mela Q., Mura M., Astara G. (2012). Randomized phase III clinical trial of a combined treatment with carnitine + celecoxib +/− megestrol acetate for patients with cancer-related anorexia/cachexia syndrome. Clin. Nutr..

[13] Mantovani G., Maccio A., Madeddu C., Serpe R., Massa E., Dessi M., Panzone F., Contu P. (2010). Randomized phase III clinical trial of five different arms of treatment in 332 patients with cancer cachexia. Oncologist.

[14] Temel J.S., Abernethy A.P., Currow D.C., Friend J., Duus E.M., Yan Y., Fearon K.C. (2016). Anamorelin in patients with non-small-cell lung cancer and cachexia (ROMANA 1 and ROMANA 2): Results from two randomised, double-blind, phase 3 trials. Lancet Oncol..

[15] Garcia J.M., Boccia R.V., Graham C.D., Yan Y., Duus E.M., Allen S., Friend J. (2015). Anamorelin for patients with cancer cachexia: An integrated analysis of two phase 2, randomised, placebo-controlled, double-blind trials. Lancet Oncol..

[16] McDonald J., Sayers J., Anker S.D., Arends J., Balstad T.R., Baracos V., Brown L., Bye A., Dajani O., Dolan R. (2023). Physical function endpoints in cancer cachexia clinical trials: Systematic Review 1 of the cachexia endpoints series. J. Cachexia Sarcopenia Muscle.

[17] Duong T., Canbek J., Birkmeier M., Nelson L., Siener C., Fernandez-Fernandez A., Henricson E., McDonald C.M., Gordish-Dressman H., Investigators C.-D. (2021). The Minimal Clinical Important Difference (MCID) in Annual Rate of Change of Timed Function Tests in Boys with DMD. J. Neuromuscul. Dis..

[18] Koynova D., Luhmann R., Fischer R. (2013). A Framework for Managing the Minimal Clinically Important Difference in Clinical Trials. Ther. Innov. Regul. Sci..

[19] Ousmen A., Touraine C., Deliu N., Cottone F., Bonnetain F., Efficace F., Bredart A., Mollevi C., Anota A. (2018). Distribution- and anchor-based methods to determine the minimally important difference on patient-reported outcome questionnaires in oncology: A structured review. Health Qual. Life Outcomes.

[20] Turner D., Schunemann H.J., Griffith L.E., Beaton D.E., Griffiths A.M., Critch J.N., Guyatt G.H. (2010). The minimal detectable change cannot reliably replace the minimal important difference. J. Clin. Epidemiol..

[21] Gamper E.M., Musoro J.Z., Coens C., Stelmes J.J., Falato C., Groenvold M., Velikova G., Cocks K., Flechtner H.H., King M.T. (2021). Minimally important differences for the EORTC QLQ-C30 in prostate cancer clinical trials. BMC Cancer.

[22] Hong F., Bosco J.L., Bush N., Berry D.L. (2013). Patient self-appraisal of change and minimal clinically important difference on the European organization for the research and treatment of cancer quality of life questionnaire core 30 before and during cancer therapy. BMC Cancer.

[23] Raman S., Ding K., Chow E., Meyer R.M., van der Linden Y.M., Roos D., Hartsell W.F., Hoskin P., Wu J.S.Y., Nabid A. (2018). Minimal clinically important differences in the EORTC QLQ-C30 and brief pain inventory in patients undergoing re-irradiation for painful bone metastases. Qual. Life Res..

[24] Bohannon R.W. (2019). Minimal clinically important difference for grip strength: A systematic review. J. Phys. Ther. Sci..

[25] Puhan M.A., Chandra D., Mosenifar Z., Ries A., Make B., Hansel N.N., Wise R.A., Sciurba F., For the National Emphysema Treatment Trial (NETT) Research Group (2011). The minimal important difference of exercise tests in severe COPD. Eur. Respir. J..

[26] Bohannon R.W., Crouch R. (2017). Minimal clinically important difference for change in 6-minute walk test distance of adults with pathology: A systematic review. J. Eval. Clin. Pract..

[27] Crosby R.D., Kolotkin R.L., Williams G.R. (2003). Defining clinically meaningful change in health-related quality of life. J. Clin. Epidemiol..

[28] Lydick E., Epstein R.S. (1993). Interpretation of quality of life changes. Qual. Life Res..

[29] Wyrwich K.W., Tierney W.M., Wolinsky F.D. (1999). Further evidence supporting an SEM-based criterion for identifying meaningful intra-individual changes in health-related quality of life. J. Clin. Epidemiol..

[30] McDonald C.M., Henricson E.K., Abresch R.T., Florence J., Eagle M., Gappmaier E., Glanzman A.M., Spiegel R., Barth J., PTC124-GD-007-DMD Study Group (2013). The 6-minute walk test and other clinical endpoints in duchenne muscular dystrophy: Reliability, concurrent validity, and minimal clinically important differences from a multicenter study. Muscle Nerve.

[31] Benaim C., Blaser S., Leger B., Vuistiner P., Luthi F. (2019). “Minimal clinically important difference” estimates of 6 commonly-used performance tests in patients with chronic musculoskeletal pain completing a work-related multidisciplinary rehabilitation program. BMC Musculoskelet. Disord..

[32] Liljequist D., Elfving B., Skavberg Roaldsen K. (2019). Intraclass correlation—A discussion and demonstration of basic features. PLoS ONE.

[33] Wynne S.C., Patel S., Barker R.E., Jones S.E., Walsh J.A., Kon S.S., Cairn J., Loebinger M.R., Wilson R., Man W.D. (2020). Anxiety and depression in bronchiectasis: Response to pulmonary rehabilitation and minimal clinically important difference of the Hospital Anxiety and Depression Scale. Chron. Respir. Dis..

[34] Anderson L.J., Lee J., Mallen M.C., Migula D., Liu H., Wu P.C., Dash A., Garcia J.M. (2021). Evaluation of physical function and its association with body composition, quality of life and biomarkers in cancer cachexia patients. Clin. Nutr..

[35] Burney B.O., Hayes T.G., Smiechowska J., Cardwell G., Papusha V., Bhargava P., Konda B., Auchus R.J., Garcia J.M. (2012). Low testosterone levels and increased inflammatory markers in patients with cancer and relationship with cachexia. J. Clin. Endocrinol. Metab..

[36] Cong M., Song C., Xu H., Song C., Wang C., Fu Z., Ba Y., Wu J., Xie C., Chen G. (2022). The patient-generated subjective global assessment is a promising screening tool for cancer cachexia. BMJ Support. Palliat. Care.

[37] Dolin T.G., Mikkelsen M.K., Jakobsen H.L., Vinther A., Zerahn B., Nielsen D.L., Johansen J.S., Lund C.M., Suetta C. (2023). The prevalence of sarcopenia and cachexia in older patients with localized colorectal cancer. J. Geriatr. Oncol..

[38] Hadzibegovic S., Porthun J., Lena A., Weinlander P., Luck L.C., Potthoff S.K., Rosnick L., Frohlich A.K., Ramer L.V., Sonntag F. (2023). Hand grip strength in patients with advanced cancer: A prospective study. J. Cachexia Sarcopenia Muscle.

[39] Ohmae N., Yasui-Yamada S., Furumoto T., Wada K., Hayashi H., Kitao M., Yamanaka A., Kubo M., Matsuoka M., Kamimura S. (2023). Muscle mass, quality, and strength; physical function and activity; and metabolic status in cachectic patients with head and neck cancer. Clin. Nutr. ESPEN.

[40] Stephens N.A., Gray C., MacDonald A.J., Tan B.H., Gallagher I.J., Skipworth R.J., Ross J.A., Fearon K.C., Greig C.A. (2012). Sexual dimorphism modulates the impact of cancer cachexia on lower limb muscle mass and function. Clin. Nutr..

[41] Perera S., Mody S.H., Woodman R.C., Studenski S.A. (2006). Meaningful change and responsiveness in common physical performance measures in older adults. J. Am. Geriatr. Soc..

[42] Bohannon R.W., Glenney S.S. (2014). Minimal clinically important difference for change in comfortable gait speed of adults with pathology: A systematic review. J. Eval. Clin. Pract..

[43] Dobs A.S., Boccia R.V., Croot C.C., Gabrail N.Y., Dalton J.T., Hancock M.L., Johnston M.A., Steiner M.S. (2013). Effects of enobosarm on muscle wasting and physical function in patients with cancer: A double-blind, randomised controlled phase 2 trial. Lancet Oncol..

[44] Solheim T.S., Laird B.J.A., Balstad T.R., Stene G.B., Bye A., Johns N., Pettersen C.H., Fallon M., Fayers P., Fearon K. (2017). A randomized phase II feasibility trial of a multimodal intervention for the management of cachexia in lung and pancreatic cancer. J. Cachexia Sarcopenia Muscle.

[45] Wright T.J., Dillon E.L., Durham W.J., Chamberlain A., Randolph K.M., Danesi C., Horstman A.M., Gilkison C.R., Willis M., Richardson G. (2018). A randomized trial of adjunct testosterone for cancer-related muscle loss in men and women. J. Cachexia Sarcopenia Muscle.

[46] Maccio A., Madeddu C., Gramignano G., Mulas C., Floris C., Sanna E., Cau M.C., Panzone F., Mantovani G. (2012). A randomized phase III clinical trial of a combined treatment for cachexia in patients with gynecological cancers: Evaluating the impact on metabolic and inflammatory profiles and quality of life. Gynecol. Oncol..

[47] Cereda E., Turri A., Klersy C., Cappello S., Ferrari A., Filippi A.R., Brugnatelli S., Caraccia M., Chiellino S., Borioli V. (2019). Whey protein isolate supplementation improves body composition, muscle strength, and treatment tolerance in malnourished advanced cancer patients undergoing chemotherapy. Cancer Med..

[48] Jatoi A., Steen P.D., Atherton P.J., Moore D.F., Rowland K.M., Le-Lindqwister N.A., Adonizio C.S., Jaslowski A.J., Sloan J., Loprinzi C. (2017). A double-blind, placebo-controlled randomized trial of creatine for the cancer anorexia/weight loss syndrome (N02C4): An Alliance trial. Ann. Oncol..

[49] Herodes M., Anderson L.J., Shober S., Schur E.A., Graf S.A., Ammer N., Salas R., Marcelli M., Garcia J.M. (2023). Pilot clinical trial of macimorelin to assess safety and efficacy in patients with cancer cachexia. J. Cachexia Sarcopenia Muscle.

[50] Kouchaki B., Janbabai G., Alipour A., Ala S., Borhani S., Salehifar E. (2018). Randomized double-blind clinical trial of combined treatment with megestrol acetate plus celecoxib versus megestrol acetate alone in cachexia-anorexia syndrome induced by GI cancers. Support. Care Cancer.

[51] Wen H.S., Li X., Cao Y.Z., Zhang C.C., Yang F., Shi Y.M., Peng L.M. (2012). Clinical studies on the treatment of cancer cachexia with megestrol acetate plus thalidomide. Chemotherapy.

[52] Hunter C.N., Abdel-Aal H.H., Elsherief W.A., Farag D.E., Riad N.M., Alsirafy S.A. (2021). Mirtazapine in Cancer-Associated Anorexia and Cachexia: A Double-Blind Placebo-Controlled Randomized Trial. J. Pain. Symptom Manag..

[53] Laviano A., Calder P.C., Schols A., Lonnqvist F., Bech M., Muscaritoli M. (2020). Safety and Tolerability of Targeted Medical Nutrition for Cachexia in Non-Small-Cell Lung Cancer: A Randomized, Double-Blind, Controlled Pilot Trial. Nutr. Cancer.

[54] Wiedenmann B., Malfertheiner P., Friess H., Ritch P., Arseneau J., Mantovani G., Caprioni F., Van Cutsem E., Richel D., DeWitte M. (2008). A multicenter, phase II study of infliximab plus gemcitabine in pancreatic cancer cachexia. J. Support. Oncol..

[55] Famil-Dardashti A., Hajigholami A., Badri S., Yekdaneh A., Moghaddas A. (2020). The role of Trigonella, Cichorium, and Foeniculum herbal combination in the treatment of cancer-induced Anorexia/Cachexia: A quasi-experimental study. Int. J. Cancer Manag..

[56] Xie M., Chen X., Qin S., Bao Y., Bu K., Lu Y. (2018). Clinical study on thalidomide combined with cinobufagin to treat lung cancer cachexia. J. Cancer Res. Ther..

[57] Gordon J.N., Trebble T.M., Ellis R.D., Duncan H.D., Johns T., Goggin P.M. (2005). Thalidomide in the treatment of cancer cachexia: A randomised placebo controlled trial. Gut.

[58] Fairman C.M., Kendall K.L., Hart N.H., Taaffe D.R., Galvao D.A., Newton R.U. (2019). The potential therapeutic effects of creatine supplementation on body composition and muscle function in cancer. Crit. Rev. Oncol. Hematol..

[59] Dans M., Kutner J.S., Agarwal R., Baker J.N., Bauman J.R., Beck A.C., Campbell T.C., Carey E.C., Case A.A., Dalal S. (2021). NCCN Guidelines(R) Insights: Palliative Care, Version 2.2021. J. Natl. Compr. Cancer Netw..

[60] Arrieta O., Cardenas-Fernandez D., Rodriguez-Mayoral O., Gutierrez-Torres S., Castanares D., Flores-Estrada D., Reyes E., Lopez D., Barragan P., Soberanis Pina P. (2024). Mirtazapine as Appetite Stimulant in Patients With Non-Small Cell Lung Cancer and Anorexia: A Randomized Clinical Trial. JAMA Oncol..

[61] Kumar N., Barai S., Gambhir S., Rastogi N. (2017). Effect of Mirtazapine on Gastric Emptying in Patients with Cancer-associated Anorexia. Indian. J. Palliat. Care.

[62] Laimer M., Kramer-Reinstadler K., Rauchenzauner M., Lechner-Schoner T., Strauss R., Engl J., Deisenhammer E.A., Hinterhuber H., Patsch J.R., Ebenbichler C.F. (2006). Effect of mirtazapine treatment on body composition and metabolism. J. Clin. Psychiatry.

[63] Roeland E.J., Bohlke K., Baracos V.E., Smith T.J., Loprinzi C.L., Cancer Cachexia Expert Panel (2023). Cancer Cachexia: ASCO Guideline Rapid Recommendation Update. J. Clin. Oncol..

[64] Roeland E.J., Bohlke K., Baracos V.E., Bruera E., Del Fabbro E., Dixon S., Fallon M., Herrstedt J., Lau H., Platek M. (2020). Management of Cancer Cachexia: ASCO Guideline. J. Clin. Oncol..

[65] Cella D., Hahn E.A., Dineen K. (2002). Meaningful change in cancer-specific quality of life scores: Differences between improvement and worsening. Qual. Life Res..

[66] Setiawan T., Sari I.N., Wijaya Y.T., Julianto N.M., Muhammad J.A., Lee H., Chae J.H., Kwon H.Y. (2023). Cancer cachexia: Molecular mechanisms and treatment strategies. J. Hematol. Oncol..

[67] Jatoi A., Ritter H.L., Dueck A., Nguyen P.L., Nikcevich D.A., Luyun R.F., Mattar B.I., Loprinzi C.L. (2010). A placebo-controlled, double-blind trial of infliximab for cancer-associated weight loss in elderly and/or poor performance non-small cell lung cancer patients (N01C9). Lung Cancer.

[68] Alley D.E., Shardell M.D., Peters K.W., McLean R.R., Dam T.T., Kenny A.M., Fragala M.S., Harris T.B., Kiel D.P., Guralnik J.M. (2014). Grip strength cutpoints for the identification of clinically relevant weakness. J. Gerontol. A Biol. Sci. Med. Sci..

[69] Cruz-Jentoft A.J., Bahat G., Bauer J., Boirie Y., Bruyere O., Cederholm T., Cooper C., Landi F., Rolland Y., Sayer A.A. (2019). Sarcopenia: Revised European consensus on definition and diagnosis. Age Ageing.

[70] Guralnik J., Bandeen-Roche K., Bhasin S.A.R., Eremenco S., Landi F., Muscedere J., Perera S., Reginster J.Y., Woodhouse L., Vellas B. (2020). Clinically Meaningful Change for Physical Performance: Perspectives of the ICFSR Task Force. J. Frailty Aging.

[71] Borg J.J., Anker S.D., Rosano G., Serracino-Inglott A., Strasser F. (2015). Multimodal management as requirement for the clinical use of anticachexia drugs—A regulatory and a clinical perspective. Curr. Opin. Support. Palliat. Care.

[72] Aryal S., Bachman S.L., Lyden K., Clay I. (2023). Measuring What Is Meaningful in Cancer Cachexia Clinical Trials: A Path Forward With Digital Measures of Real-World Physical Behavior. JCO Clin. Cancer Inform..

[73] Blum D., Vagnildhaug O.M., Stene G.B., Maddocks M., Sorensen J., Laird B.J.A., Prado C.M., Skeidsvoll Solheim T., Arends J., Hopkinson J. (2023). Top Ten Tips Palliative Care Clinicians Should Know About Cachexia. J. Palliat. Med..

[74] Garcia J.M., Dunne R.F., Santiago K., Martin L., Birnbaum M.J., Crawford J., Hendifar A.E., Kochanczyk M., Moravek C., Piccinin D. (2022). Addressing unmet needs for people with cancer cachexia: Recommendations from a multistakeholder workshop. J. Cachexia Sarcopenia Muscle.

[75] Hopkinson J.B. (2023). Educational needs of self-care in cachectic cancer patients and caregivers. Curr. Opin. Oncol..

[76] Norman K., Stobaus N., Reiss J., Schulzke J., Valentini L., Pirlich M. (2012). Effect of sexual dimorphism on muscle strength in cachexia. J. Cachexia Sarcopenia Muscle.

[77] Wright A., Hannon J., Hegedus E.J., Kavchak A.E. (2012). Clinimetrics corner: A closer look at the minimal clinically important difference (MCID). J. Man. Manip. Ther..

[78] Antoun S., Raynard B. (2018). Muscle protein anabolism in advanced cancer patients: Response to protein and amino acids support, and to physical activity. Ann. Oncol..

[79] Dalton J.T., Barnette K.G., Bohl C.E., Hancock M.L., Rodriguez D., Dodson S.T., Morton R.A., Steiner M.S. (2011). The selective androgen receptor modulator GTx-024 (enobosarm) improves lean body mass and physical function in healthy elderly men and postmenopausal women: Results of a double-blind, placebo-controlled phase II trial. J. Cachexia Sarcopenia Muscle.

[80] Evans M., Guthrie N., Pezzullo J., Sanli T., Fielding R.A., Bellamine A. (2017). Efficacy of a novel formulation of L-Carnitine, creatine, and leucine on lean body mass and functional muscle strength in healthy older adults: A randomized, double-blind placebo-controlled study. Nutr. Metab..

[81] Ruiz-Garcia V., Lopez-Briz E., Carbonell-Sanchis R., Bort-Marti S., Gonzalvez-Perales J.L. (2018). Megestrol acetate for cachexia-anorexia syndrome. A systematic review. J. Cachexia Sarcopenia Muscle.

[82] Lim Y.L., Teoh S.E., Yaow C.Y.L., Lin D.J., Masuda Y., Han M.X., Yeo W.S., Ng Q.X. (2022). A Systematic Review and Meta-Analysis of the Clinical Use of Megestrol Acetate for Cancer-Related Anorexia/Cachexia. J. Clin. Med..

[83] Katakami N., Uchino J., Yokoyama T., Naito T., Kondo M., Yamada K., Kitajima H., Yoshimori K., Sato K., Saito H. (2018). Anamorelin (ONO-7643) for the treatment of patients with non-small cell lung cancer and cachexia: Results from a randomized, double-blind, placebo-controlled, multicenter study of Japanese patients (ONO-7643-04). Cancer.

[84] Takayama K., Katakami N., Yokoyama T., Atagi S., Yoshimori K., Kagamu H., Saito H., Takiguchi Y., Aoe K., Koyama A. (2016). Anamorelin (ONO-7643) in Japanese patients with non-small cell lung cancer and cachexia: Results of a randomized phase 2 trial. Support. Care Cancer.

[85] Granger C.L., Holland A.E., Gordon I.R., Denehy L. (2015). Minimal important difference of the 6-minute walk distance in lung cancer. Chron. Respir. Dis..

[86] Holland A.E., Hill C.J., Rasekaba T., Lee A., Naughton M.T., McDonald C.F. (2010). Updating the minimal important difference for six-minute walk distance in patients with chronic obstructive pulmonary disease. Arch. Phys. Med. Rehabil..

[87] Tager T., Hanholz W., Cebola R., Frohlich H., Franke J., Doesch A., Katus H.A., Wians F.H., Frankenstein L. (2014). Minimal important difference for 6-minute walk test distances among patients with chronic heart failure. Int. J. Cardiol..

[88] Mathai S.C., Puhan M.A., Lam D., Wise R.A. (2012). The minimal important difference in the 6-minute walk test for patients with pulmonary arterial hypertension. Am. J. Respir. Crit. Care Med..

[89] Smith S.M., Dworkin R.H., Turk D.C., McDermott M.P., Eccleston C., Farrar J.T., Rowbotham M.C., Bhagwagar Z., Burke L.B., Cowan P. (2020). Interpretation of chronic pain clinical trial outcomes: IMMPACT recommended considerations. Pain.

